# System dynamics analysis on the effectiveness of vaccination and social mobilization policies for COVID-19 in the United States

**DOI:** 10.1371/journal.pone.0268443

**Published:** 2022-08-12

**Authors:** Jiayi Chen, Shuo-Yan Chou, Tiffany Hui-Kuang Yu, Zakka Ugih Rizqi, Dinh Thi Hang

**Affiliations:** 1 Department of Public Finance, Feng Chia University, Taichung, Taiwan, ROC; 2 Department of Industrial Management, National Taiwan University of Science and Technology, Taipei, Taiwan, ROC; Centers for Disease Control and Prevention, UNITED STATES

## Abstract

The COVID-19 pandemic has presented significant public health and economic challenges worldwide. Various health and non-pharmaceutical policies have been adopted by different countries to control the spread of the virus. To shed light on the impact of vaccination and social mobilization policies during this wide-ranging crisis, this paper applies a system dynamics analysis on the effectiveness of these two types of policies on pandemic containment and the economy in the United States. Based on the simulation of different policy scenarios, the findings are expected to help decisions and mitigation efforts throughout this pandemic and beyond.

## 1. Introduction

The *Coronavirus Disease 2019* (COVID-19) pandemic has affected daily life, the global economy, and our public health systems worldwide [1]. COVID-19 can spread with alarming speed, mainly through person-to-person contact. According to Johns Hopkins University CSSE COVID-19 [2], as of 25 May 2021, the global estimate is 167,011,807 cases including 3,472,068 deaths. Current hotspots are in the Americas, Europe, and South-East Asia, where the majority are developed countries. Even though these countries have the most advanced healthcare systems in the world, they have faced an increase in daily infections and deaths [3]. Another point worth noting is the challenge presented by COVID-19 variants. Under suitable conditions, a virus will mutate into different strains which may be more harmful or resistant to medical treatment. This is yet another burden on the global health system, which already faces shortages of pharmaceuticals and critical medical supplies, as well as trade-off of rapid vaccine production [4]. No doubt, COVID-19 is a large burden on the public health system currently [5]. Hence, the pandemic has had and will likely continue to have negative impact on the global economy, socio-system, and health system [6].

The pandemic has spread quickly and widely, straining the world economy in ways unseen for decades [7]. The three main aspects of this strain are: production productivity disruption, supply chain and market disruption, and financial recession [8]. Many countries have implemented strict lockdowns, placing restrictions on movement and encouraging social distancing. Governments have struggled to mitigate the negative effects of fragmentation of global trade and supply linkages, labor shortages, and shutdowns [9]. The combination of lockdowns restricting movement and people reducing expenses has dealt a stinging blow to many industries: retail, travel, restaurants, etc. [10]. Multiple companies around the world are on the brink of bankruptcy. Further, the pandemic has put pressure on subdued capital flows and tight financial conditions amid mounting debt.

From the socio-economic perspective, people’s livelihoods have been severely affected, especially for the most vulnerable groups such as low-income citizens, the elderly, persons with disabilities, children, and women [11, 12]. Many struggle in finding gainful employment. Jobs often offer only low and irregular income and are frequently in high-risk environments which increase the risk of spreading the virus to themselves, their families, and their communities [13]. The United Nations has found that around 70% of women in developing countries have fallen out of employment, worked in unsafe conditions, or took a break from work to take care of their children and family [14]. Apart from physical suffering, the consequences of quarantine and isolation measures have affected mental health [15]. Despite all this, governments should encourage their citizens to perform self-isolation and pandemic prevention.

Currently, the vaccine is considered to be a promising way to reduce transmission, infections, and deaths due to COVID-19 as well as an essential means to end the pandemic. With the extraordinary success in vaccine development, there have been more than 292 vaccine candidates and over 70 have entered clinical evaluation, allowing governments to consider vaccination strategies [16, 17]. Whereas traditional development can take 15 years or longer, the development time for SAR-CoV 2 was significantly reduced [18]. However, there is a need to address issues of trust in vaccines and related institutions to achieve rapid global immunization [19]. Vaccine distribution is faced with logistical and political problems such as the disparity in distribution between high- or upper-middle-income countries and low-income countries [20]. To illustrate, higher-income countries account for around 82% of developed doses, whereas for poorer countries have received only 0.2% of available vaccines. 25% of people have received the vaccine across higher-income countries, while the ratio falls to 1 in 500 among lowest-income countries [21]. In the first half of 2021, Israel, Canada, and the United Kingdom have emerged as pioneers countries regarding vaccination programs, with the share of people who received at least one dose at around 63%, 59%, and 58.6%, respectively [22]. Vaccine distribution will continue to play a critical role in bringing an end to the pandemic.

The United States (US) has launched an ambitious nationwide vaccination campaign. Around 138 million people, accounting for 44.88% of the population, are fully vaccinated [23]. In the US, three vaccine providers: Pfizer-BioNTech, Moderna, and Johnson & Johnson vaccines have received Emergency Use Authorization (EUA) for active immunization to prevent COVID-19 from the US Food and Drug Administration (FDA). Pfizer and Moderna vaccines have been reported as having strong efficacy against the virus [24], rated at 95% and 94%, respectively [25]. The US makes for an excellent case study to assess the impact of vaccinations, including the social impact to other variables in a complex system. Besides that, data from the US on COVID-19 is reliable—information is published for public view.

The severity of the pandemic situation has been an attractive topic for researchers in various fields such as public health [20, 26]; economics [27, 28]; societal [29]; and education [30]. Qualitative and quantitative models have been used to obtain a better understanding of disease outbreaks, response, and policy of governments worldwide [31]. A study analyzing endogenous testing, containment measures, and social distancing [32–35] used the system dynamics model to figure out the high uncertainty in the parameters of the infectious disease model. Some studies in utilizing system dynamics have been applied to assess COVID-19 by considering the social subsystem only [36–42]. Other studies have tried to consider the economic subsystem since COVID-19 has burdened the economy of the country [33, 34, 43, 44]. Different from previous studies, this study will incorporate a vaccination subsystem into a system dynamics framework since vaccination is the only way to achieve herd immunity against COVID-19. To the best of our knowledge, vaccination has not been addressed in system dynamics literature for solving COVID-19. Therefore, this is our significant contribution in terms of model extension and policy design development in modeling COVID-19 pandemic.

This study provides a system dynamics analysis using the U.S. as a case study with three main purposes: (i) develop system dynamics framework for COVID-19 modeling by considering vaccination, economics, and social mobilization subsystems; (ii) use system dynamics model to assess the effect of vaccination supply and isolation to other subsystems; (iii) propose potential recommendations for the U.S. to decrease the number of infected people and economic burden due to COVID-19. Moreover, the proposed model also can be generalized to assess vaccination policy in other countries. The rest of this paper is organized as follows: Section 2 discusses the result of literature and comparison between previous researches with ours. Section 3 presents the proposed system dynamics model under the following three subsystems: economics subsystem, social subsystem, and vaccine subsystem. The assumptions and validation stage are also described in this section. Section 4 details the experimental design of system dynamics model and alternative selection. Finally, the conclusions as well as future ideas are provided in Section 5.

## 2. Literature review

### 2.1. System dynamics modeling

System Dynamics (SD) is defined as a methodology for understanding how things change over time by focusing on the feedback (loop) behavior of variables within the systems and deals with complex systems as well as non-linearity and delay [45]. It was proposed by MIT professor Jay Forrester in the mid-1950s. The strength of system dynamics lies in the way it analyses the impact of system feedback in a complex system by considering the uncertainty simultaneously. It follows the system thinking paradigm as the basic principle [46]. System thinking sees the system component as a stock acting as an accumulator representing a quantity at specific time and the flow acting as a rate changing stock value measured over an interval of time. System thinking also sees the relation of a system component as circular not linear. Therefore, feedback can be captured and the whole system can be assessed systematically.

Generally, system dynamics follows two stages of modeling, conceptual modeling and computer simulation modeling [47]. Conceptual modeling establishes a mental model of dynamic problems that are presented using Causal Loop Diagrams (CLD). Further, variables identified in CLD are translated in terms of stocks and flows (a so-called flow diagram) between the stocks as well as the information determining the value of the flows [48] in the computerized model using simulation software such as Stella, Powersim, and Vensim where differential equations are the main drivers of the model [49].

System dynamics is very useful when creating highly abstract simulations of complex systems, especially social engineering [50, 51] through computer simulation technology. SD can be used to get insight about human behavior and contribute to understanding of social systems for behavioral forecasting [52]. This capability is especially useful to model the situations around COVID-19 and generate helpful insight. There are three reasons for using SD to model the situations arising from the COVID-19 pandemic [13]. First, the paradigm of SD supports the possibility of using complex cause-and-effect relationships and dependencies, including reinforcing and balancing feedback loops as a main characteristic of society in a complex system. Second, the incubation period of the infection can be modelled via SD in the form of a delay. Finally, the SD paradigm enables using probability distribution to model the system in the absence of complete and accurate information to determine system indicators.

### 2.2. Compartment model

To model an epidemic problem [53], proposed the SIR Compartment Model which stands for Susceptible—Infected—Recovered to reduce the complexity of mathematical models or infectious disease. In this model, it is assumed that the number of individuals belonging to a particular group change over time according to the specified characteristics of dependencies. In other words, S+I+R will be equal to the population number at a time (t). This model uses differential equations.

The rate of transition of individuals from the susceptible partition to the infected partition is affected by the frequency of contact between individuals in the population, the infectiousness of the disease, and the number of susceptible and infected individuals at the present time [54]. The SIR model assumes that all recovered patients have permanent immunity to the disease, so that all recovered people will not be infected again.

This analysis makes some modifications to the SIR model. The modifications allow for an enriched model to follow the conditions of the pandemic. One of the modifications is the SIS model (Susceptible—Infected—Susceptible). It is used to simulate the spread of diseases if affected individuals are not immune to reinfection [55]. There is also the Susceptible—Infected—Recovered—Susceptible (SIRS) model. This model is used if recovered individuals obtain immunity, but gradually lose it due to virus mutations or others factors [56]. The Susceptible—Exposed—Infected—Recovered (SEIR) model adds an exposed group which contains infected people who are asymptomatic or in incubation period [57].

Of all the models, the SIR and SEIR models are the ones most often used in COVID-19 research [58]. There are also two extended versions of the SIR and SEIR models which are the SIRD and SEIRD models. These two models add a Dead partition to simulate the mortality of individuals as a result of disease [59]. Experimental research has proven that both models are efficient tools for modeling and forecasting the future situations of the COVID-19 pandemic [60]. However, it is not easy to obtain and divide data between the exposed and infected partitions since asymptomatic and symptomatic patients are not easy to differentiate. Therefore, this paper uses the SIRD model. The differential equations of the SIRD model used are shown in Eqs 1–4.


$$\frac{dS}{dt}=-\beta \;I\left(t\right)\;S\left(t\right)$$ (1)

$$\frac{dI}{dt}=\beta \;I\left(t\right)\;S\left(t\right)-\left(\gamma +\mu \right)\;I\left(t\right)$$ (2)

$$\frac{dR}{dt}=\gamma \;I\left(t\right)$$ (3)

$$\frac{dD}{dt}=\mu \;I\left(t\right)$$ (4)

These equations describe the mathematical model to calculate how people pass from one partition to another. Susceptible (S) people become infected (I) by the virus, after that they either die (D) or recover (R). In other words, the equations above can be understood to mean that every partition will be derived by time (t) based on the constant. For example, Eq 4 means that the variation of the number of deaths will be proportional to the infected individuals, with the proportionality constant being denoted by μ. The constant γ is the recovery rate and β is the constant of infected rate. The sum of all partitions at (t) will have the constant (N) which represents the total number of people. Eq 1 has a minus sign because it will subtract the number of susceptible people (S).

### 2.3. Research on COVID-19 with system dynamics modeling

There are some existing research papers related to System Dynamics Modeling for the COVID-19 pandemic. A summary is shown in Table 1. To the best of our knowledge, there is still no study related to System Dynamics modeling for the COVID-19 pandemic by considering a vaccination subsystem. There has been no research yet which tries to consider policies related to vaccine supply and what effect such policies may have on social and economic subsystems of a country. Moreover, vaccination is becoming the main solution to help protect people from infection. This is our main contribution in terms of variables considered and policy design development related to the COVID-19 pandemic.

**Table 1:** Related research to COVID-19 pandemic.

Authors	Country	Subsystem			Policy Design				
		Social	Economic	Vaccination	Isolation	Vaccine Supply	Social Distance	Public Health	Testing
[34]	Philippines	**✓**	**✓**	-	**✓**	-	**✓**	**✓**	**-**
[33]	New Mexico	**✓**	**✓**	-	**✓**	-	**✓**	-	**✓**
[36]	South Africa	**✓**	-	-	**✓**	-	**✓**	**-**	**-**
[43]	Philippines	**✓**	**✓**	-	**✓**	-	**✓**	**✓**	**-**
[37]	Malaysia	**✓**	-	-	-	-	**✓**	-	-
[38]	Thailand	**✓**	-	-	-	-	**✓**	-	-
[44]	Russia	**✓**	**✓**	-	**✓**	-	-	-	-
[39]	China	**✓**	-	-	-	-	**✓**	-	-
[40]	Japan	**✓**	-	-	**✓**	-	**✓**	-	-
[41]	China	**✓**	-	-	-	-	**✓**	-	-
[42]	Kenya	**✓**	-	-	**✓**	-	**✓**	-	-
Own	United States	**✓**	**✓**	**✓**	**✓**	**✓**	**-**	**-**	**-**

## 3. Methods

### 3.1. Model structure

System dynamics is a method of system modeling and dynamic simulation, which can be used to analyze the dynamic complexity of social economy, environmental protection, and industrial development. There is a close dependence between the behavior of the system and the internal mechanism, and the cause-and-effect feedback relationship is interlinked as well.

Stocks and Flows Diagram (SFD) is a graphical representation of the feedback form and control law of the system and distinguishes the variables’ differences. Stock and flow are also called level and rate respectively. The system dynamic structure contains several main variables. The level variable describes the cumulative effect of the system, and its value can be measured instantaneously. The rate variable describes the change of the cumulative effect of the system over time and represents the speed of change or amplitude of a decision. The auxiliary variable is the intermediate variable in the process of information transfer and conversion between the state variable and the rate variable, which runs through the whole decision-making process. The SFD for this analysis is mainly composed of three subsystems, including the economic subsystem, social subsystem, and vaccine subsystem.

**Fig 1:**
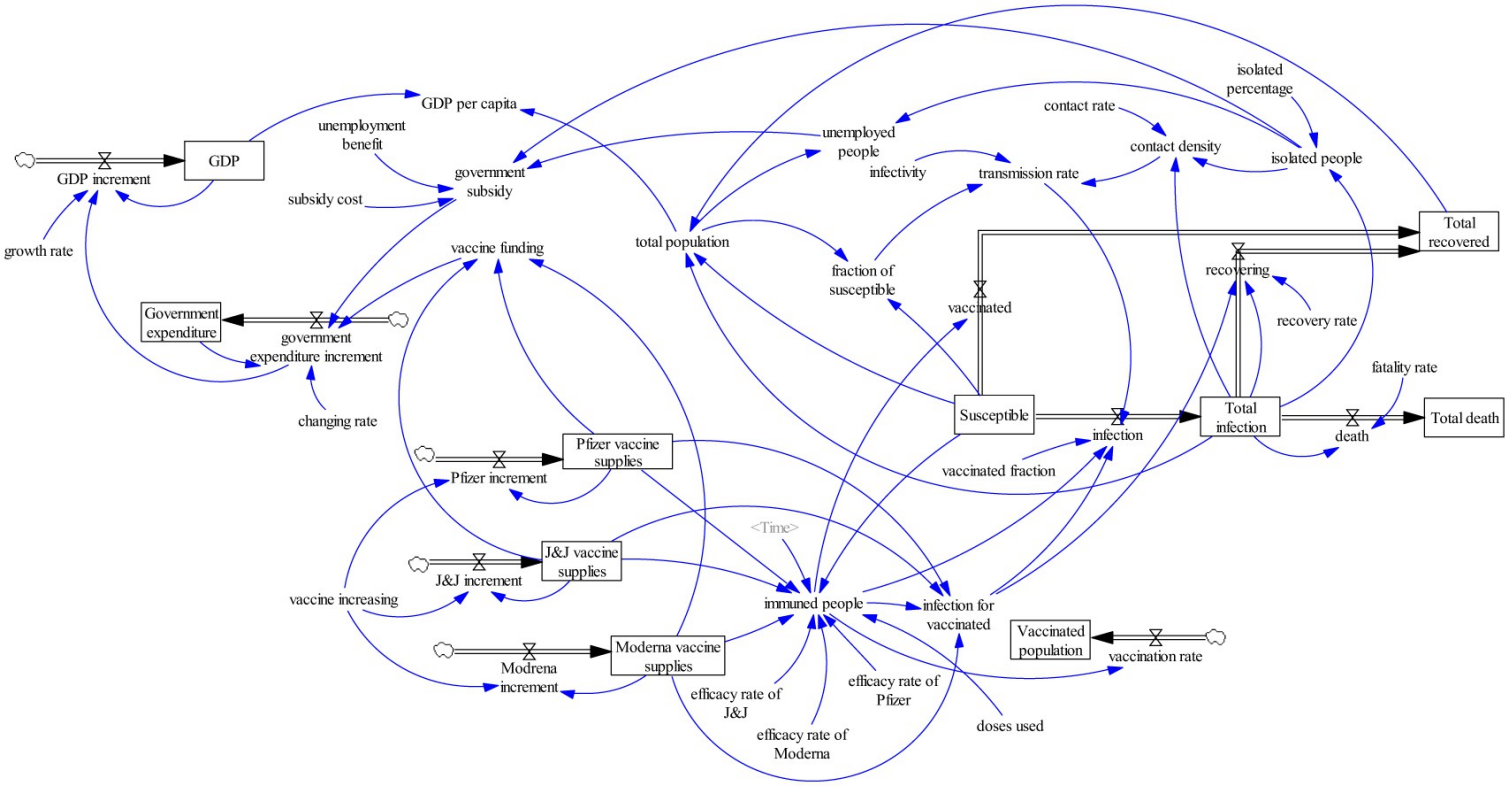
Proposed system dynamics framework of Covid-19 in the U.S.

Using the general structure of the SIRD model, we outlined the progression of the COVID-19 pandemic through different pathways under different interventions. The purpose of this study is to discuss the effect of vaccination on disease transmission and the recovery of infected persons since the outbreak of COVID-19 in the United States, as well as non-pharmaceutical interventions on unemployment and government subsidies for the pandemic. This paper analyzes the challenges faced by the three subsystems of the global pandemic and establishes the status quo of the role played by vaccine development and supply in pandemic response. From the perspective of system dynamics, the feedback relationship between the three subsystems is explored, the relationship between the internal variables is clarified, and the operation mode and trajectory of each subsystem are described (Fig 1). For simulation purpose, the data for March 2021 was validated and simulated for the next 60 days.

### 3.2. Theoretical framing analysis

#### 3.2.1. Vaccination subsystem

There are currently three main vaccines for COVID-19 in the United States: Pfizer-BioNTech, Moderna, and Johnson & Johnson (J&J). Pfizer and Moderna vaccines were available in the U.S. starting in December 2020, and J&J was approved on February 26, 2021 [61]. The effectiveness of the vaccine against the virus is a key factor shaping the current pandemic response. In this study, we hypothesized that the population vaccinated with either one or two doses was moderately effective against COVID-19. According to a new study provided by the Centers for Disease Control and Prevention (CDC) [62], people who received full doses of Moderna and Pfizer will not spread the virus with both vaccines considered around 95% effective and J&J lower than that. Those rates will be described as the efficacy rate in system dynamics model. The efficacy rate helps to model the condition seen in reality where vaccinated people may have a chance to be reinfected. In the US, more than 344 million doses have been distributed, with over 70% of doses used [63]. After warning for months that vaccinated people should still be cautious to not infect others, the CDC now suggests that those who are fully vaccinated may not be at much risk of transmitting COVID-19 [62]. The willingness of people to vaccinate is an important influencing factor, so the government’s publicity on vaccination is paramount to the pandemic response. Vaccination is an economic and effective way to prevent and control the spread of COVID-19.

#### 3.2.2. Economic subsystem

The 2020–2021 coronavirus pandemic caused severe economic disruptions last year, with varying degrees of impact on various industries. Households, governments, and businesses implemented various mandatory and voluntary measures to limit human-to-human interactions that could spread the virus, causing unemployment to rise. Job losses were concentrated among low-wage workers.

The U.S. government treats the purchase of vaccines and grants for outbreaks as emergency funds, and congress allocates the budget subject to sequestration. Part of the funding will be used by the Department of Health and Human Services to develop and maintain the pandemic response as it continues to develop.

#### 3.2.3. Social subsystem

Let’s consider the ‘primary flow’ of the virus. Susceptible people become infected through contact with an infectious person. These patients will develop symptoms, and transfer to the total infection partition. After treatment, some patients will recover and, unfortunately, some patients will become critically ill and die. The protection rate against COVID-19 death in the vaccinated population is about 66% to 72%. Our study assumes that those who recovered or passed were not vectors of transmission. If those with mild symptoms come into contact with others, they can continue to spread the virus. The implementation of an isolation policy can effectively reduce the frequency of contact with others as well as the risk of transmission, but will in turn affect normal life and work, leading to the unemployment of some people. The government will then come up with policies to deal with the social and economic impact of the COVID-19 pandemic.

### 3.3. Assumptions of the model

The SIRD model assumes:

1. A homogenous and static population hence the effects of new births and immigration are excluded. This is also supported since so many countries impose lockdown policy including United States.
2. The model does not describe the causal relationship directly about how recovered people will be re-infected. However, the possibility of reinfection still can be considered through efficacy rate inherent to the vaccine types.
3. The first dose of the vaccine will be assumed to prevent severe cases of COVID-19 and protect from infection and death.
4. Infected people will isolate (self-quarantine) as much as possible and high levels of people isolating will require government response in the form of policies and subsidies.
5. The only vaccines considered are the three most popular in the U.S.: Pfizer, Monderna, and Johnson & Johnson.
6. Vaccine related costs are the U.S. government’s responsibility.
7. The relative data of GDP are official forecasts from the Congressional Budget Office.
8. Variants of COVID-19 are not considered.

### 3.4. Model validation

**Fig 2:**
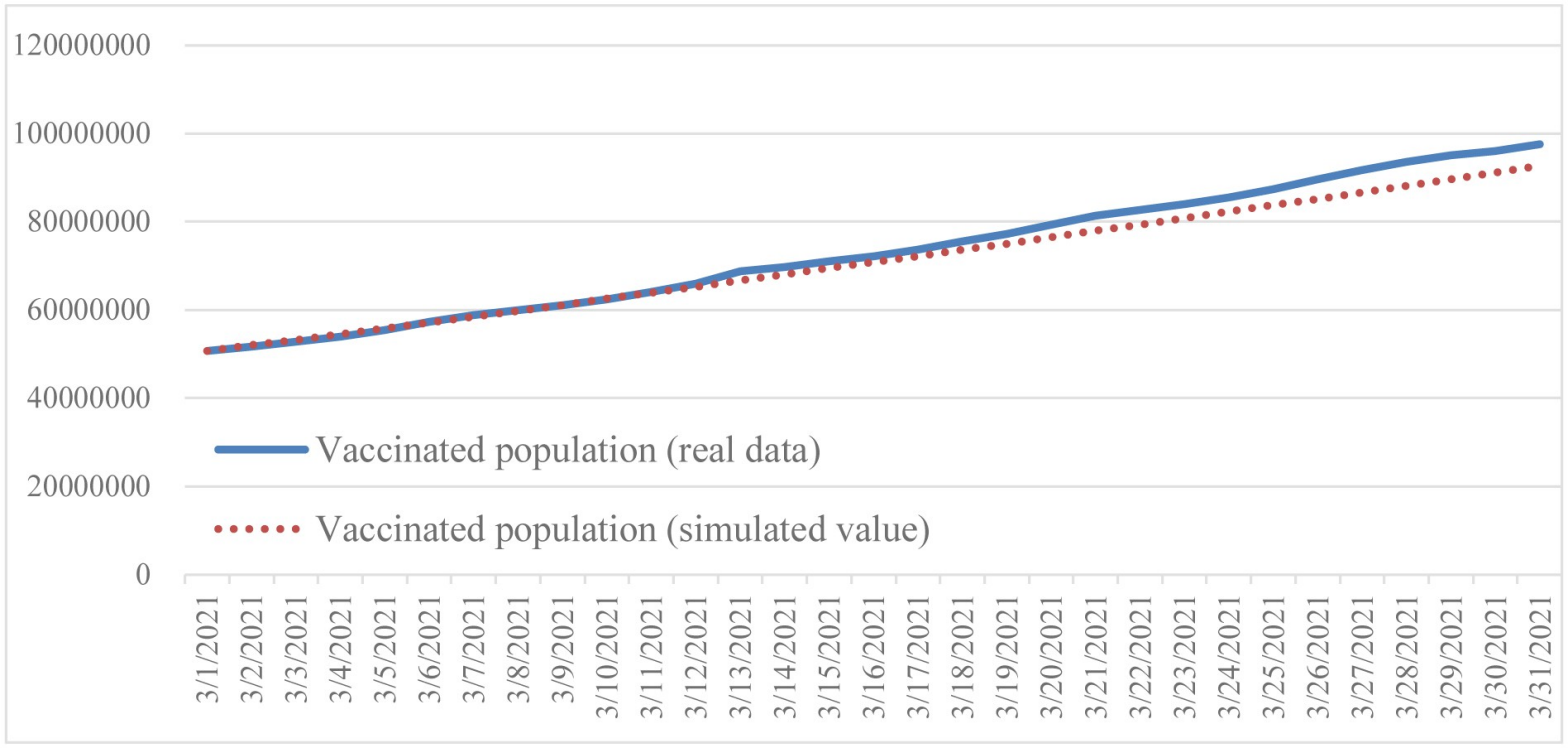
Comparison of reported vaccinated population and simulated vaccinated population.

**Fig 3:**
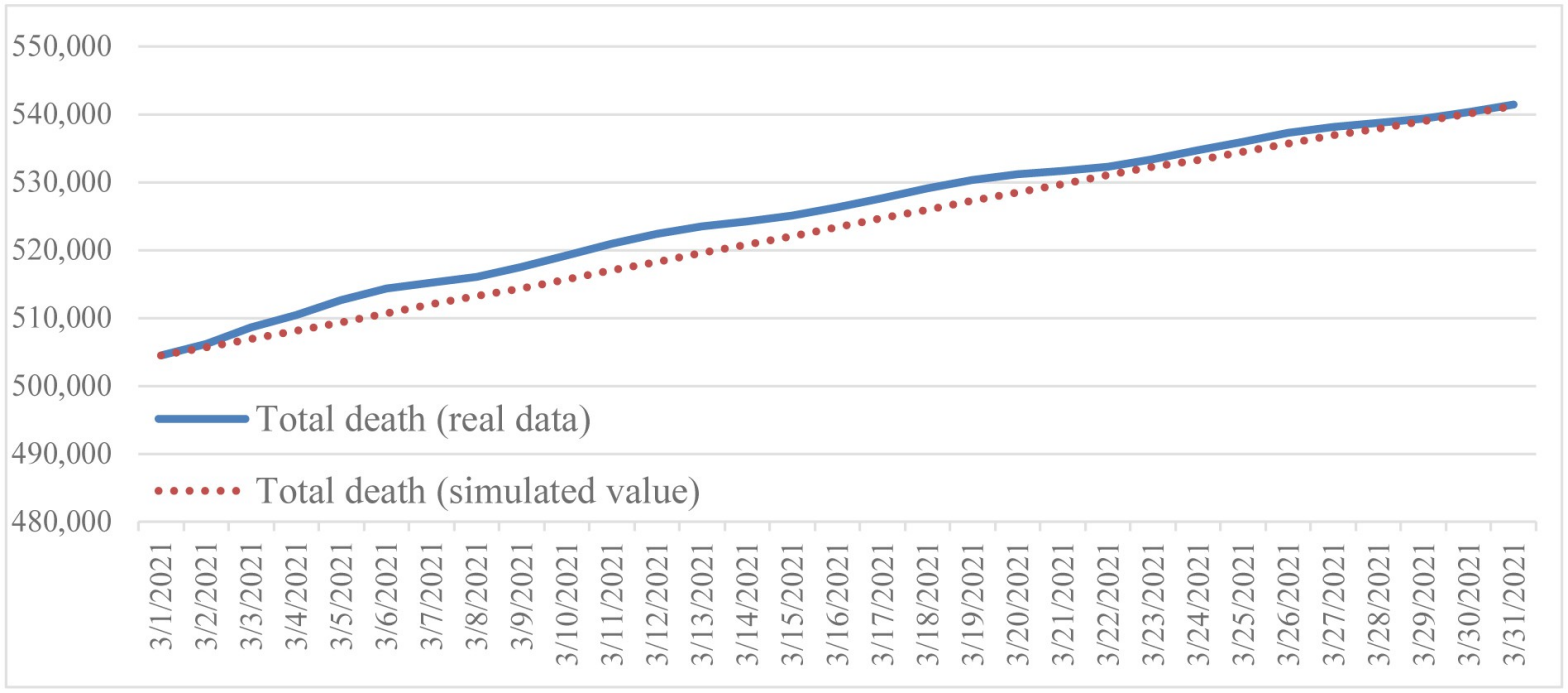
Comparison of reported total death and simulated total death.

**Fig 4:**
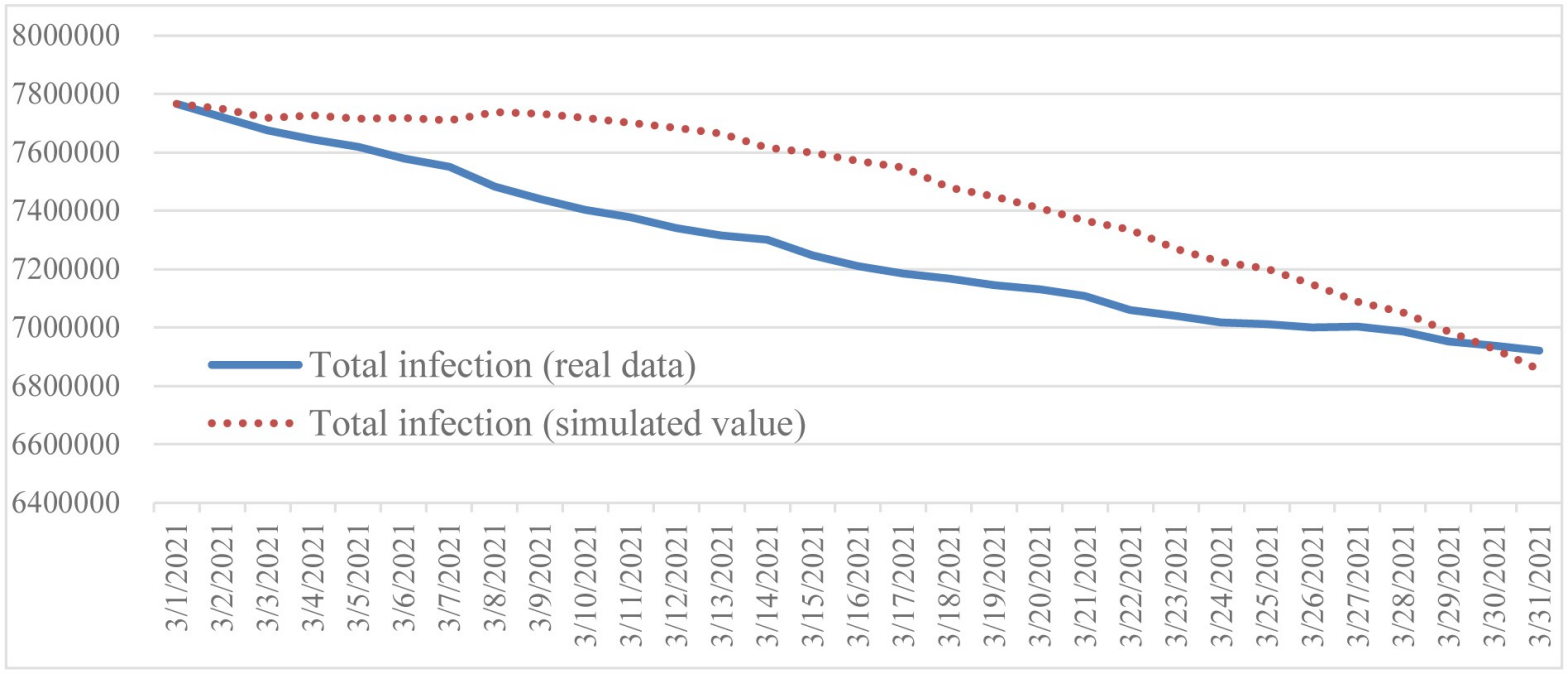
Comparison of reported total infection and simulated total infection.

This simulation is based on the following settings: the initial time is March 1, 2021, the time step is 1, and the unit is a day. The purpose of validation is to prove the reliability and credibility of the model, and to evaluate the ability of the model to reproduce historical data. For historical verification, this study selected vaccinated population, total death, and total infection as the primary variables, and compared the historical data with the model simulation results (Figs 2–4).

Based on Barlas [64], a system dynamics model will be valid if the error rate is less than 5%. Since system dynamics simulates over time, Mean Absolute Percentage Error (MAPE) is used to calculate the error rate. The equations are shown in Eqs 5 and 6, withN being number of data, A actual data, and S simulation data. The result is shown in Table 2. All MAPE values show that system dynamics fulfills validation criteria with an error rate of less than 5%. Also found is that the overall variation trend is consistent when the simulation is run under the basic conditions and scenarios. Therefore, the simulation results are acceptable and the SIRD model can provide reasonable output behavior.


$$\bar{S}=\frac{1}{N}\overset{N}{\underset{i=1}{\sum }}Si$$ (5)

$$\bar{A}=\frac{1}{N}\overset{N}{\underset{i=1}{\sum }}Ai$$ (6)

**Table 2:** Validation results.

Variables	Simulation ($\overset{-}{S}$)	Actual data ($\overset{-}{A}$)	MAPE
Vaccinated Population	73124154	71135965	2.5%
Total Death	525435	523218	0.423%
Total Infection	7269620	7467081	2.793%

## 4. Results

### 4.1. Scenario settings

The study performed model adjustments by varying auxiliary variables associated with various interventions in COVID-19 management, including isolated percentage and vaccine supply increasing per day (Table 3). The first scenario policy set in this study was simulated by changing isolated percentage separately, and 8% was taken as the baseline scenario. In real life, people are often unwilling to be isolated all the time, which will have negative effects on both economy and life. Therefore, 2% is used as Case A1 for simulation in this study. However, increasing the proportion is supposed to help to mitigate the spread of infection, thus forming Case A2. In Case B1, vaccine supply doubled per day that of the baseline scenario, and a higher proportion of 1.8% was used as Case B2 for simulation. The third scenario setting is to simulate and simultaneously change the influence of isolated percentage and vaccine supply increasesas the pandemic response responds to COVID-19 developments in combination with the previous two situations.

**Table 3:** Scenario settings under different isolated percentage and vaccine supply increasing.

	Case A			Case B			Case C				
Scenario	Base	A1	A2	Base	B1	B2	Base	C1	C2	C3	C4
Isolated percentage	8%	2%	20%	8%	8%	8%	8%	2%	2%	20%	20%
Vaccine supply increasing	0.5%	0.5%	0.5%	0.5%	1%	1.8%	0.5%	1%	1.8%	1%	1.8%

### 4.2. Change in isolated percentage: Scenario A

**Fig 5:**
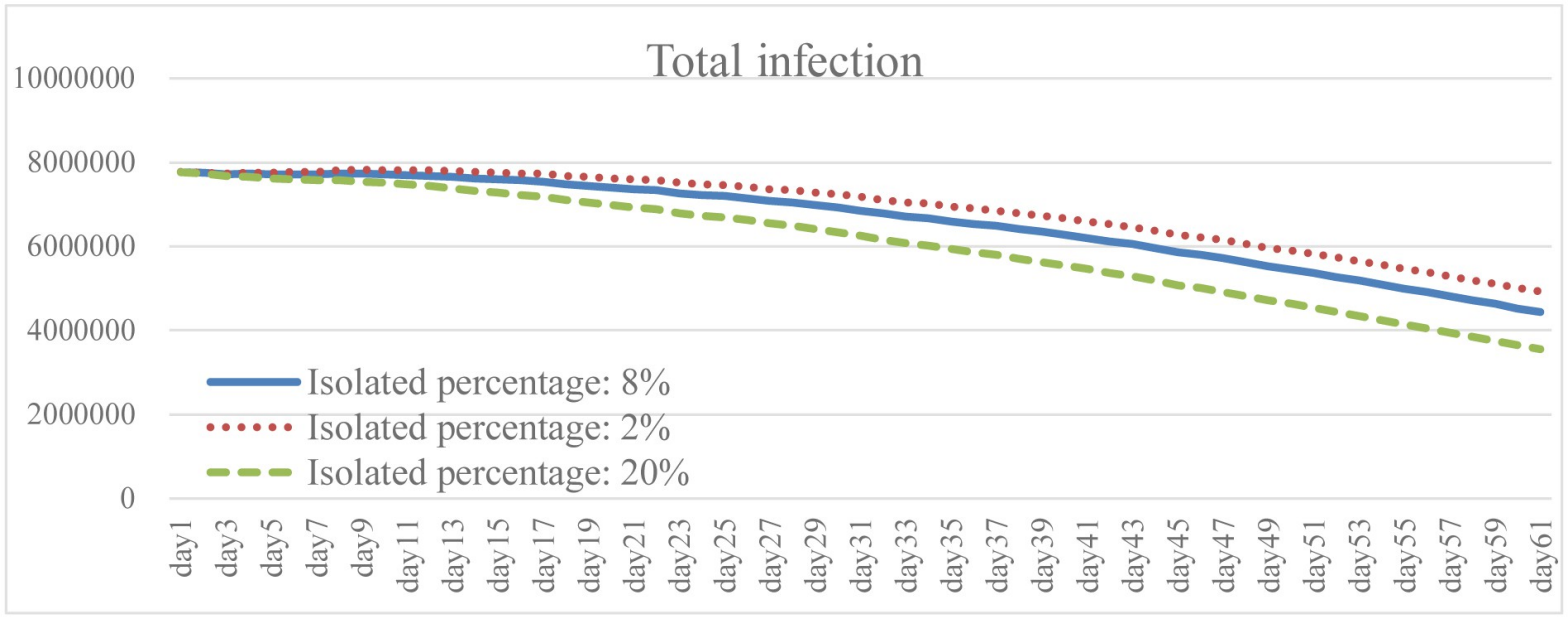
Total infection under different isolation percentage.

**Fig 6:**
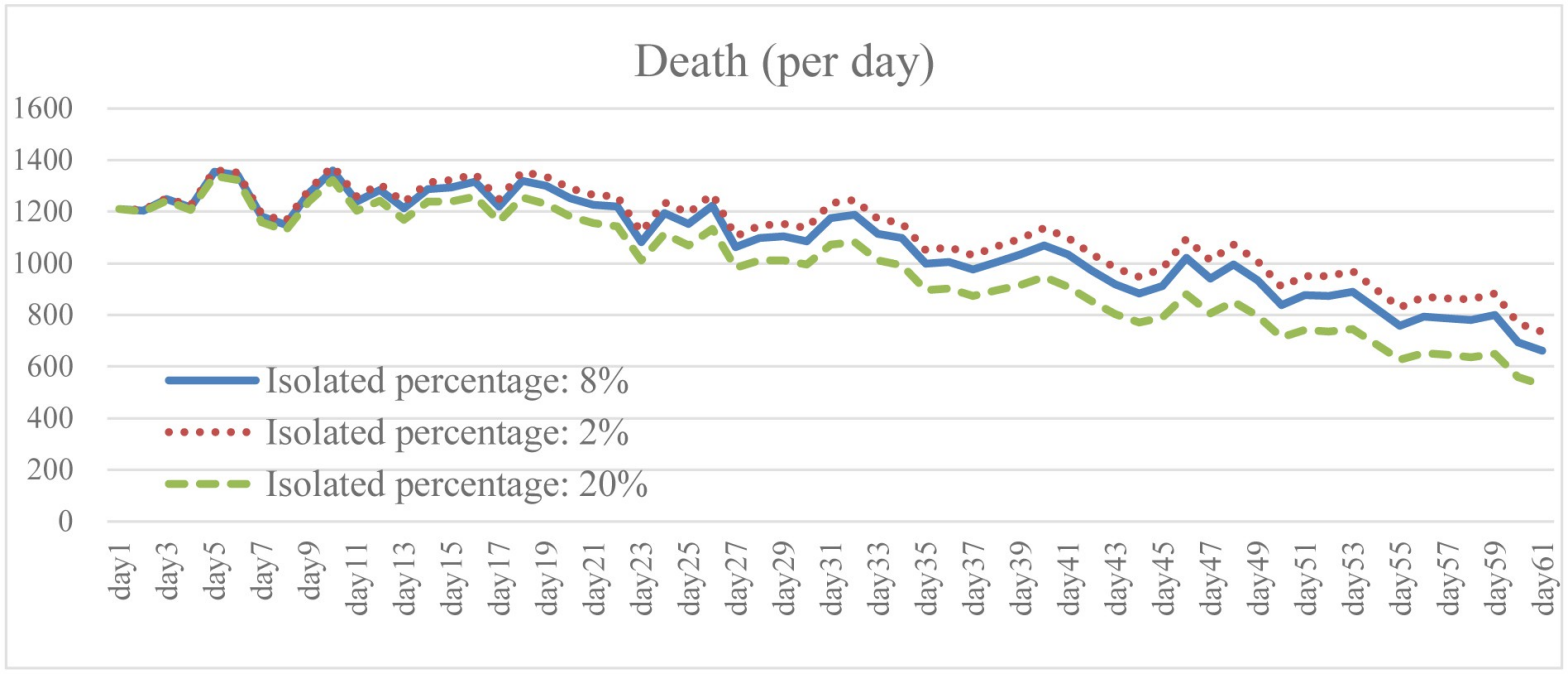
Daily death under different isolation percentage.

**Fig 7:**
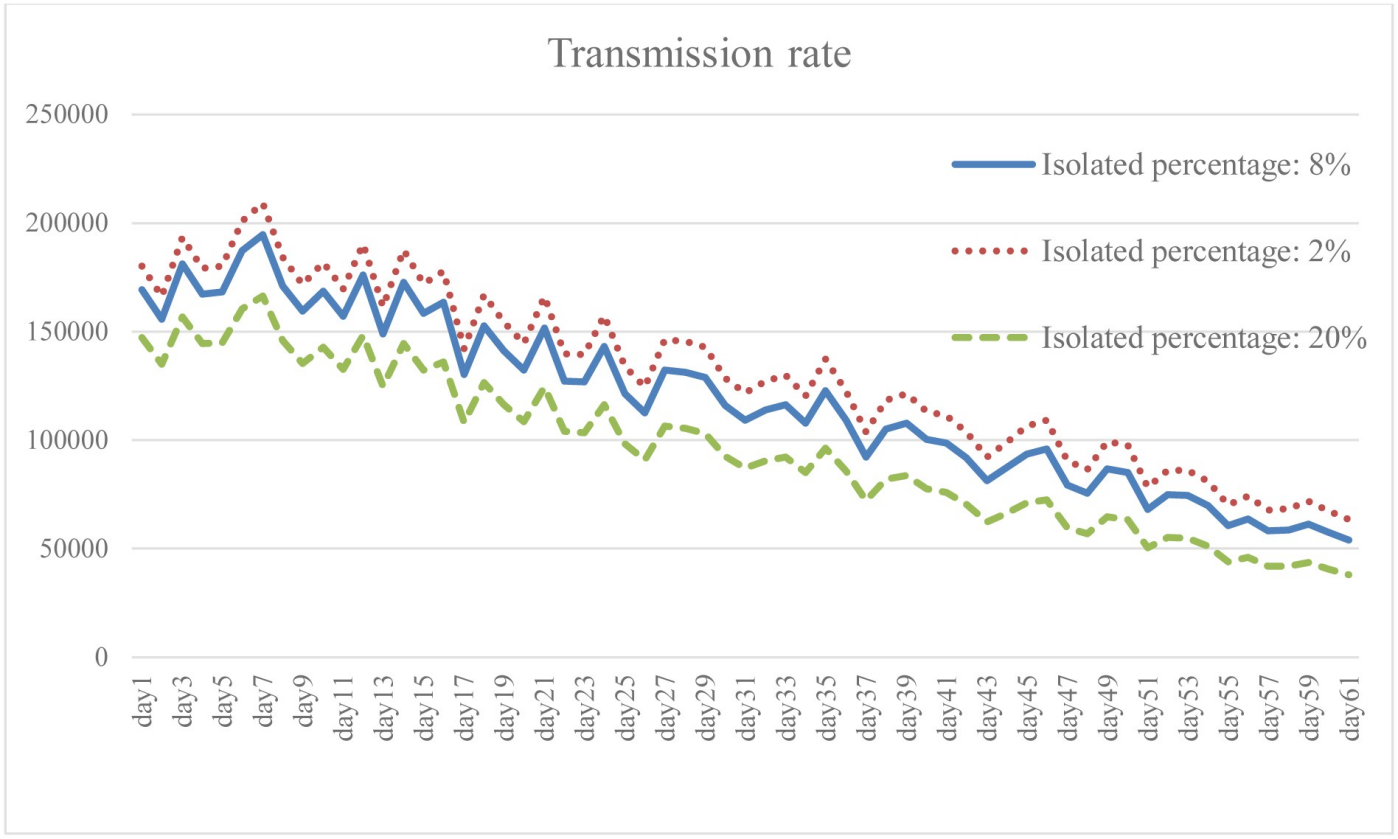
Transmission rate under different isolation percentage.

**Fig 8:**
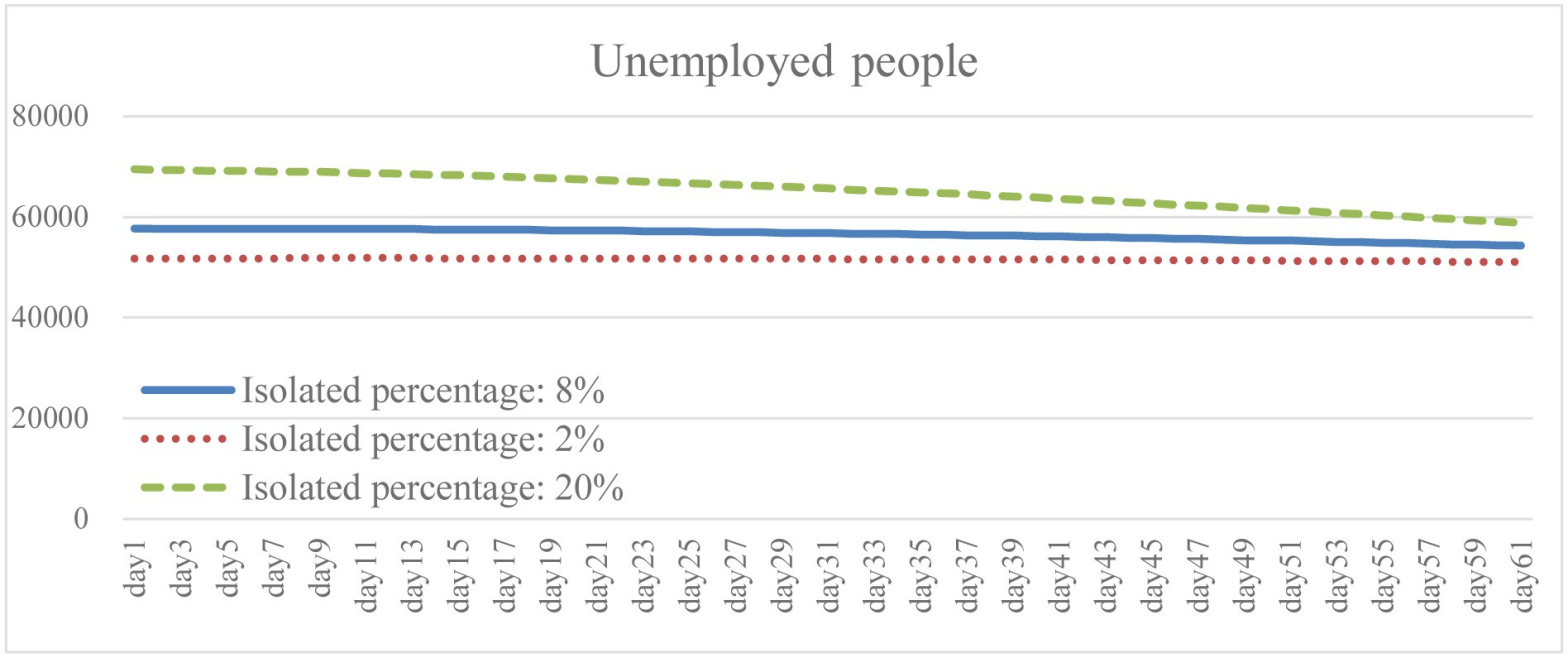
Unemployed people under different isolation percentage.

**Fig 9:**
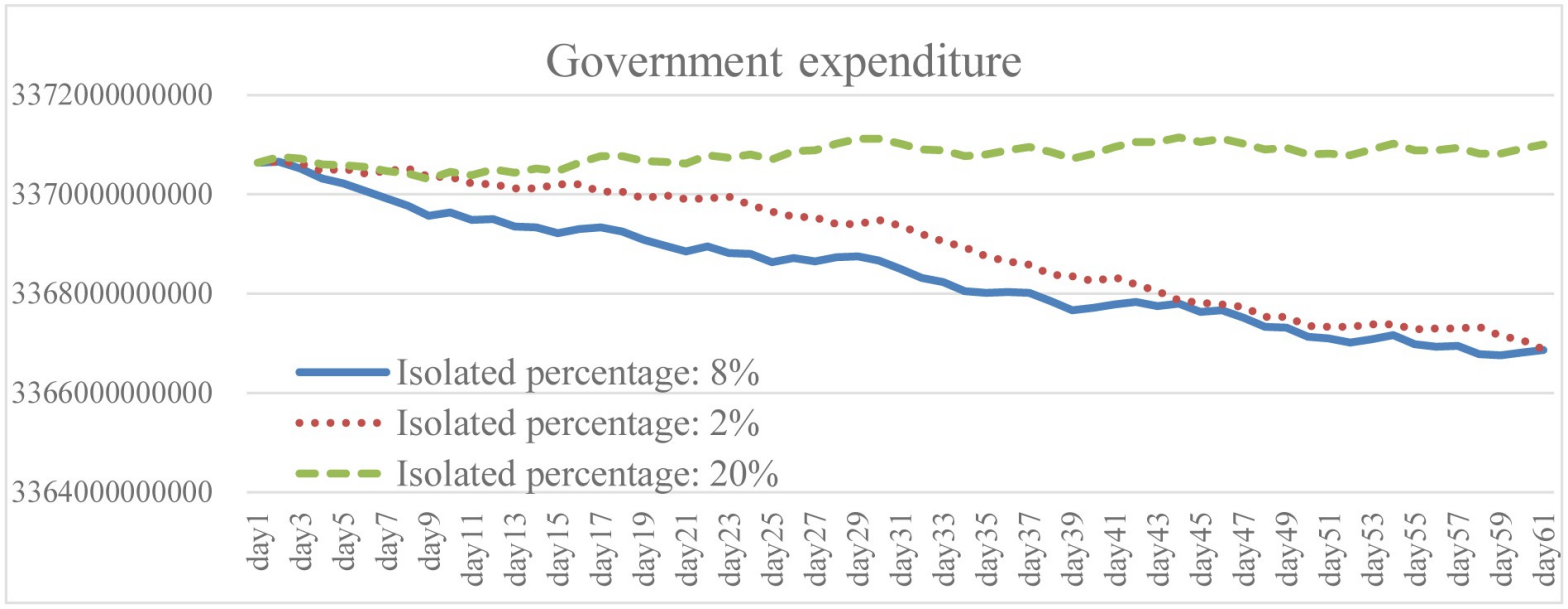
Government expenditure under different isolation percentage.

Isolation keeps someone who is infected with the virus away from others, even in their home. When the isolation rate was increased to 2.5 times the baseline, both the total number of infections and the daily death rate were well reduced as shown in the results (Figs 5 and 6). The transmission rate also decreased by an average of 20.6% (Fig 7). As the isolation rate is increased, some workers will lose their jobs. According to the simulation results, the number of unemployed in the early stage will be 18.4% higher than that in the baseline (Fig 8). However, as the measures of the pandemic response are implemented and the overall social environment is improved, the result is closer to the baseline. In addition, the federal government has introduced relevant policies to subsidize self-quarantined and the unemployed. Increasing the isolation rate will increase the financial burden of the government (as shown in Fig 9). From Fig 9, it is also known that the reduction of isolation percentage from 8% to 2% does not give an expected result. This happens due to uncertainty of some variables related to government expenditure such as changing rate, growth rate of GDP, etc. (see the S1 Appendix). This phenomenon gives a sign that the decrement of 6% is not a tipping point (small changes in parameters are not significant enough to cause an effect due to uncertainty of other variables). At the same time, increasing the isolation rate will cause much inconvenience in daily life. Most people cannot fully follow recommendations for isolation, which will lead to increases in the infection rate and death rate. If people do not actively vaccinate, COVID-19 will continue to have a detrimental impact on daily life in the United States.

### 4.3. Change in vaccine supply increase: Scenario B

**Fig 10:**
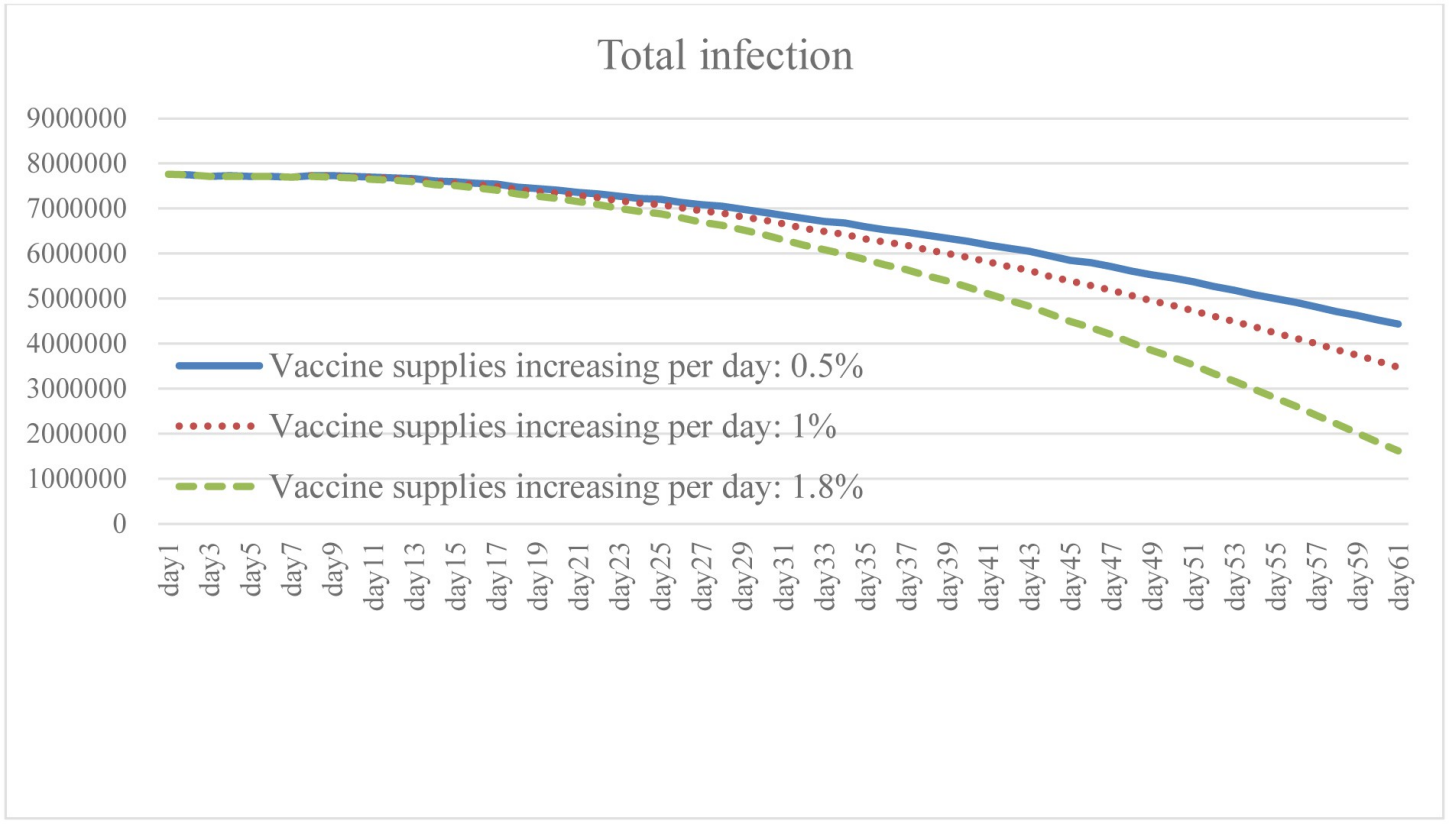
Total infection under different vaccine supply.

**Fig 11:**
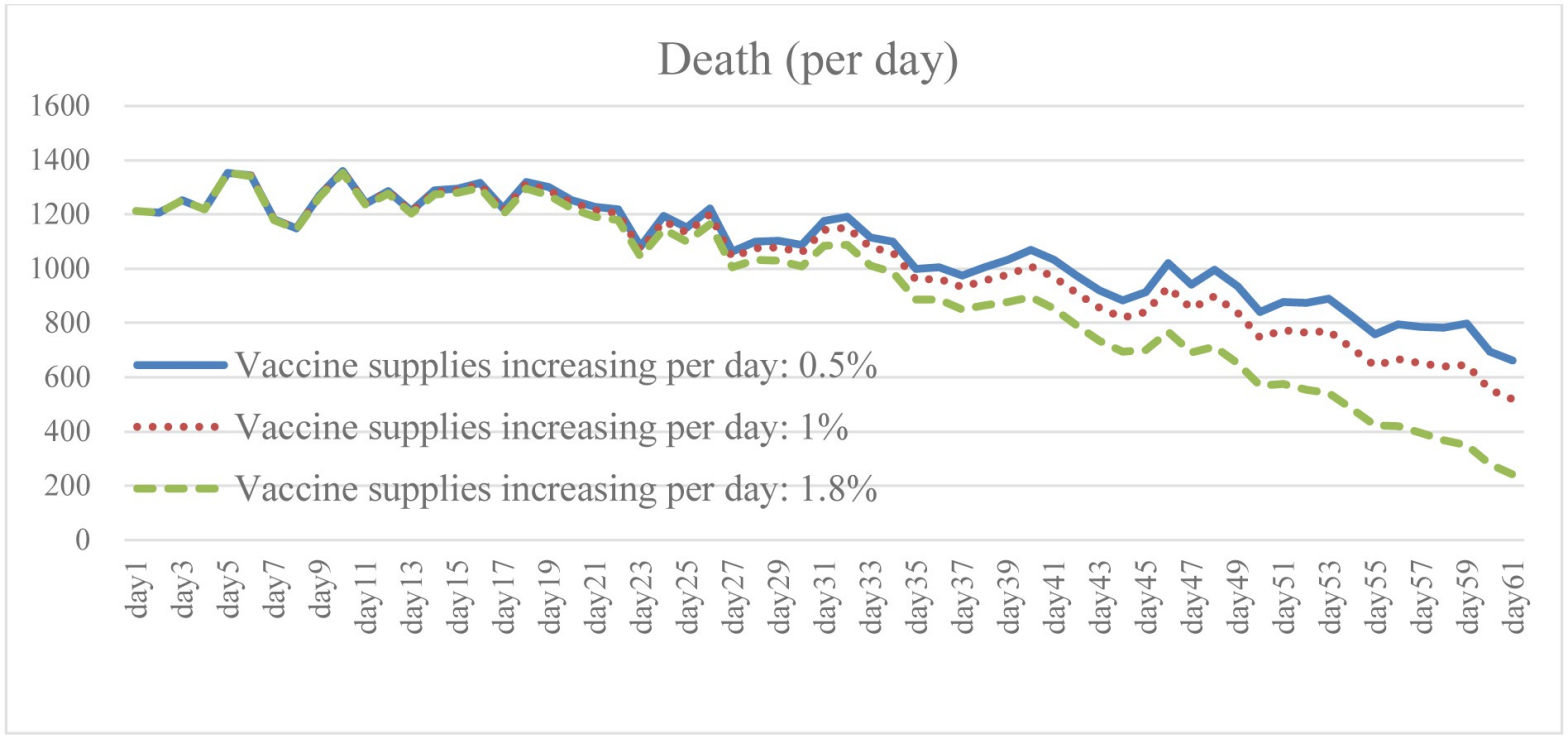
Daily death under different vaccine supply.

**Fig 12:**
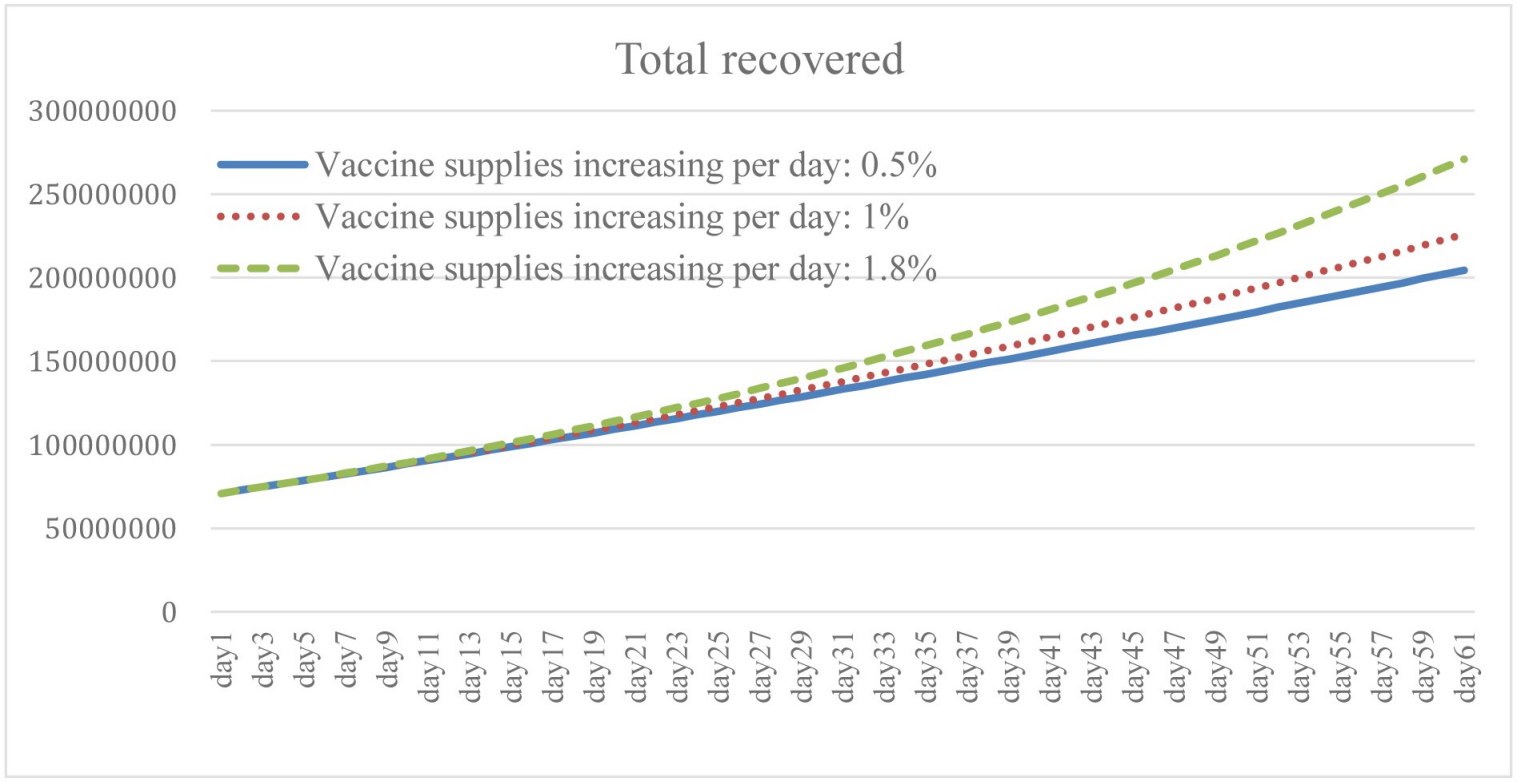
Total recovered under different vaccine supply.

**Fig 13:**
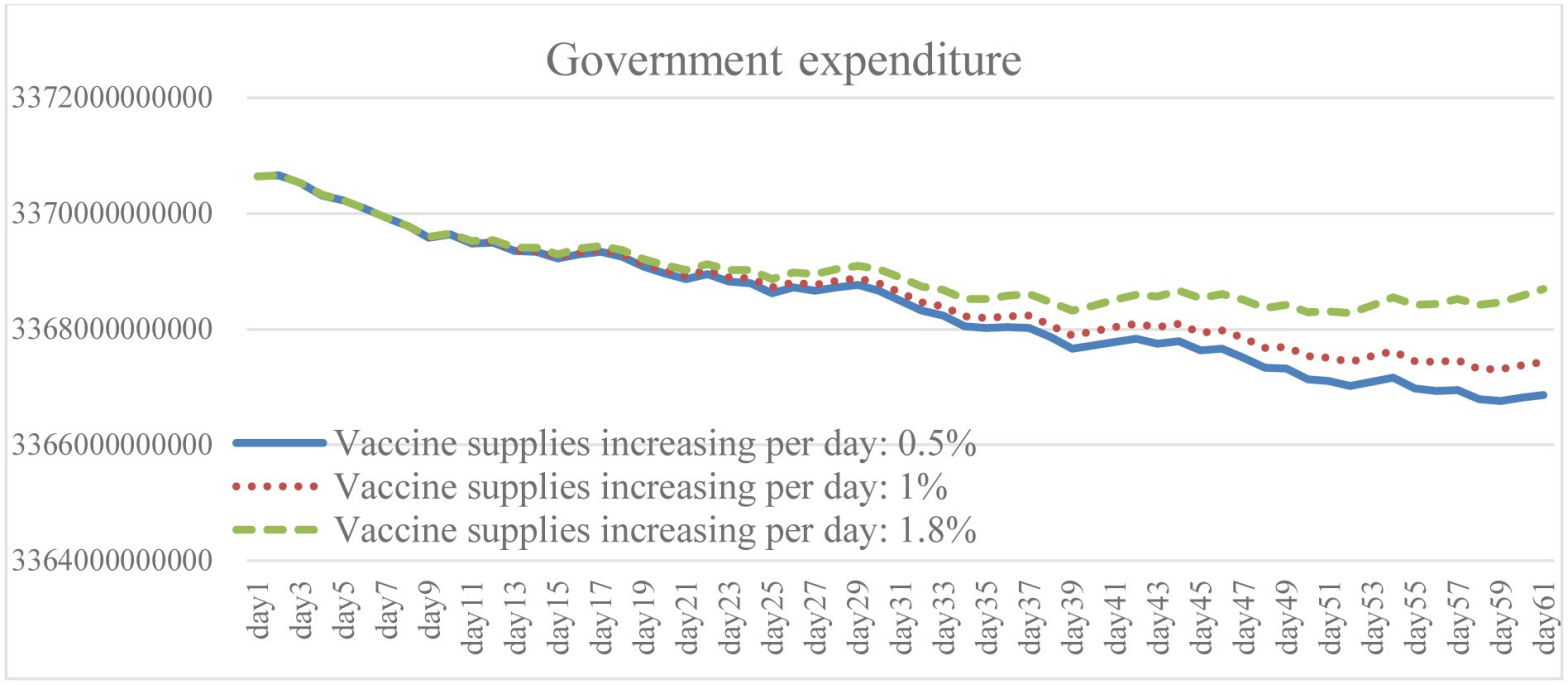
Government expenditure under different vaccine supply.

**Fig 14:**
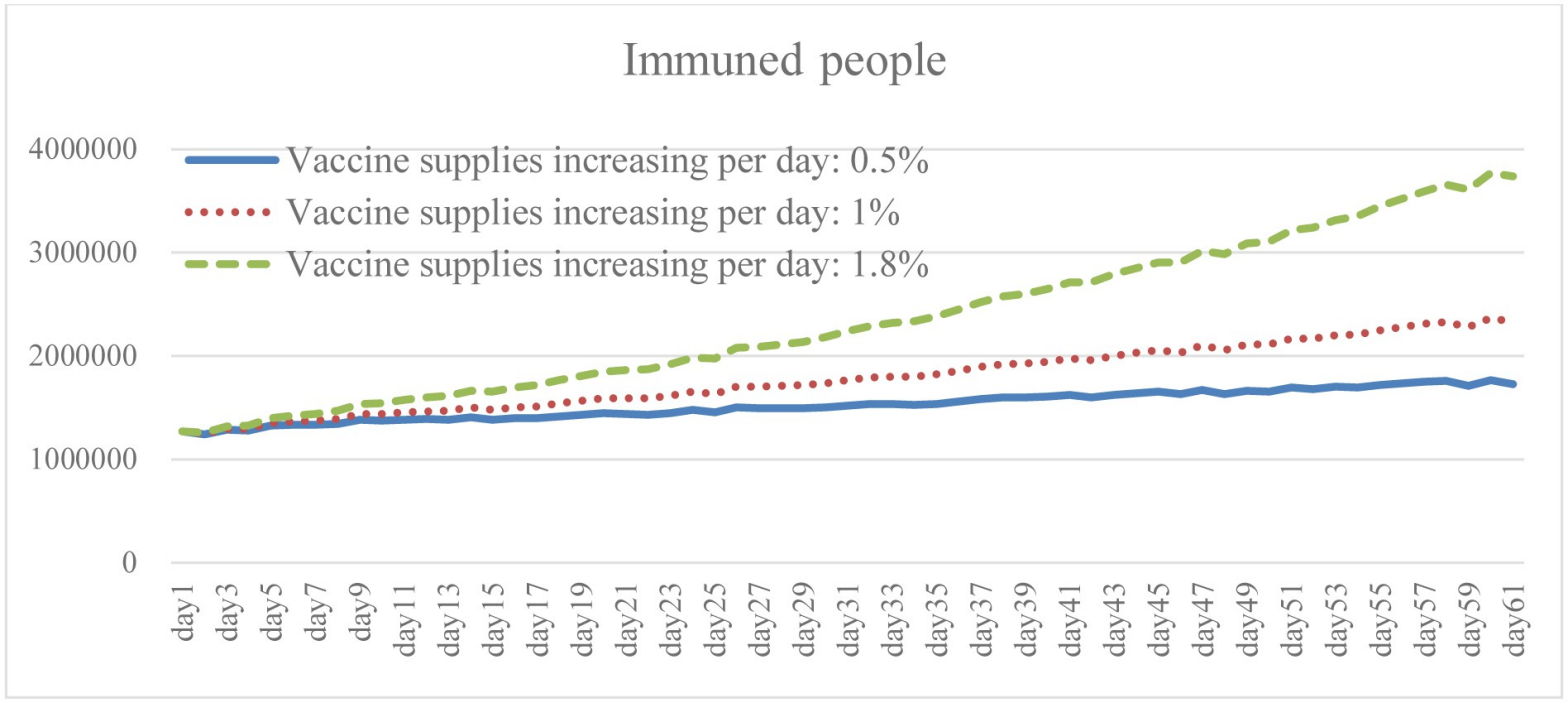
Immuned people under different vaccine supply.

**Fig 15:**
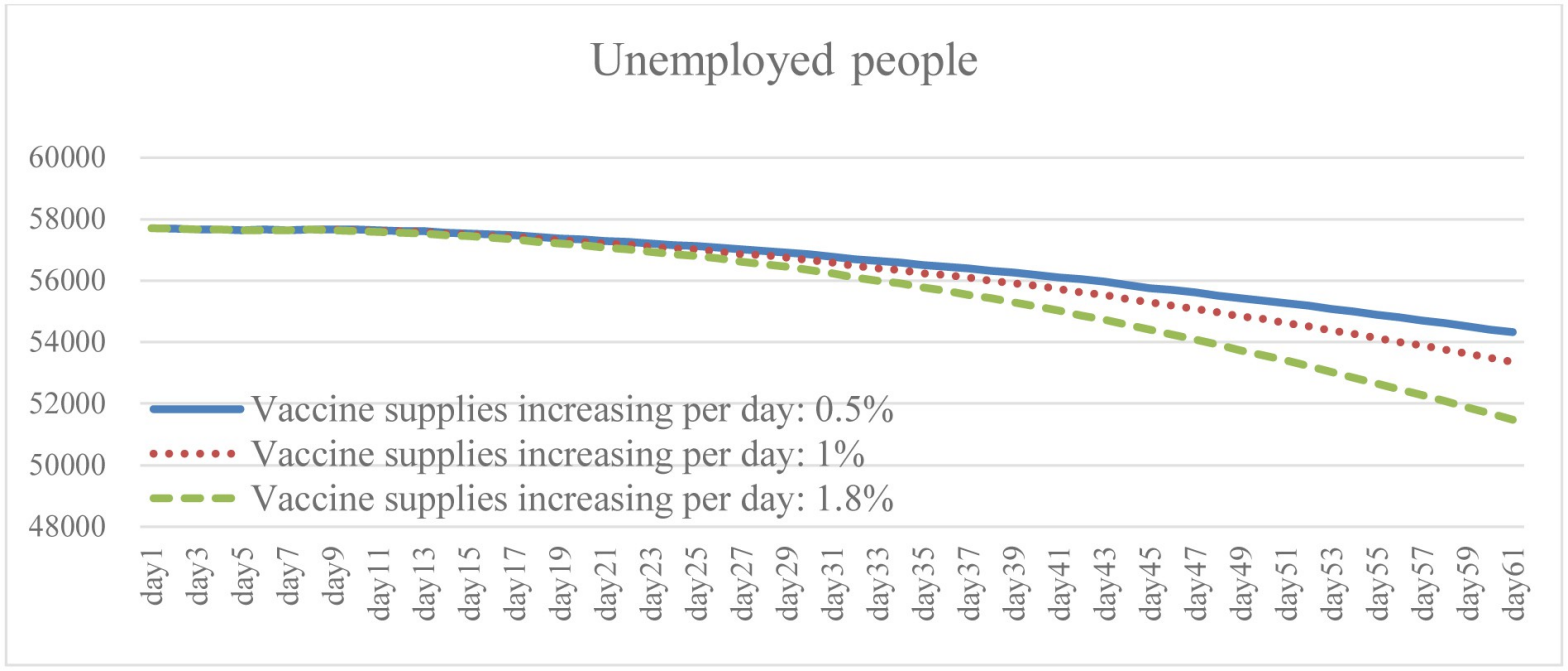
Unemployed peple under different vaccine supply.

Ensuring broad access to the COVID-19 vaccine is necessary to prevent cases and deaths, and to promote herd immunity while having the capacity to effectively respond to the pandemic. It is clear from the simulation results that the higher the growth rate of vaccines provided by the government, and of people willing to be vaccinated, the lower the total number of daily infections and death (Figs 10 and 11). The results of Case B2 decrease by 15.6% on average compared with the baseline, and the total number of recoveries increases by 11.8% (Fig 12). Since it is the government’s responsibility to provide vaccine support (in the United States), vaccine supplies are plentiful and government spending will rise to some extent (Fig 13). When the immunized population (Fig 14) is gradually expanded and the overall pandemic is effectively under control, there are signs of improvement in society and the economy as a whole, with the pressure on the unemployed alleviated (Fig 15). Government vaccine supply is important, but, at the same time, relevant pandemic prevention, vaccination, and public relations materials should be strengthened so as to improve the willingness of people to get vaccinated. Only after herd immunity is achieved can the development of COVID-19 be better controlled.

### 4.4. Combination policy: Scenario C

**Fig 16:**
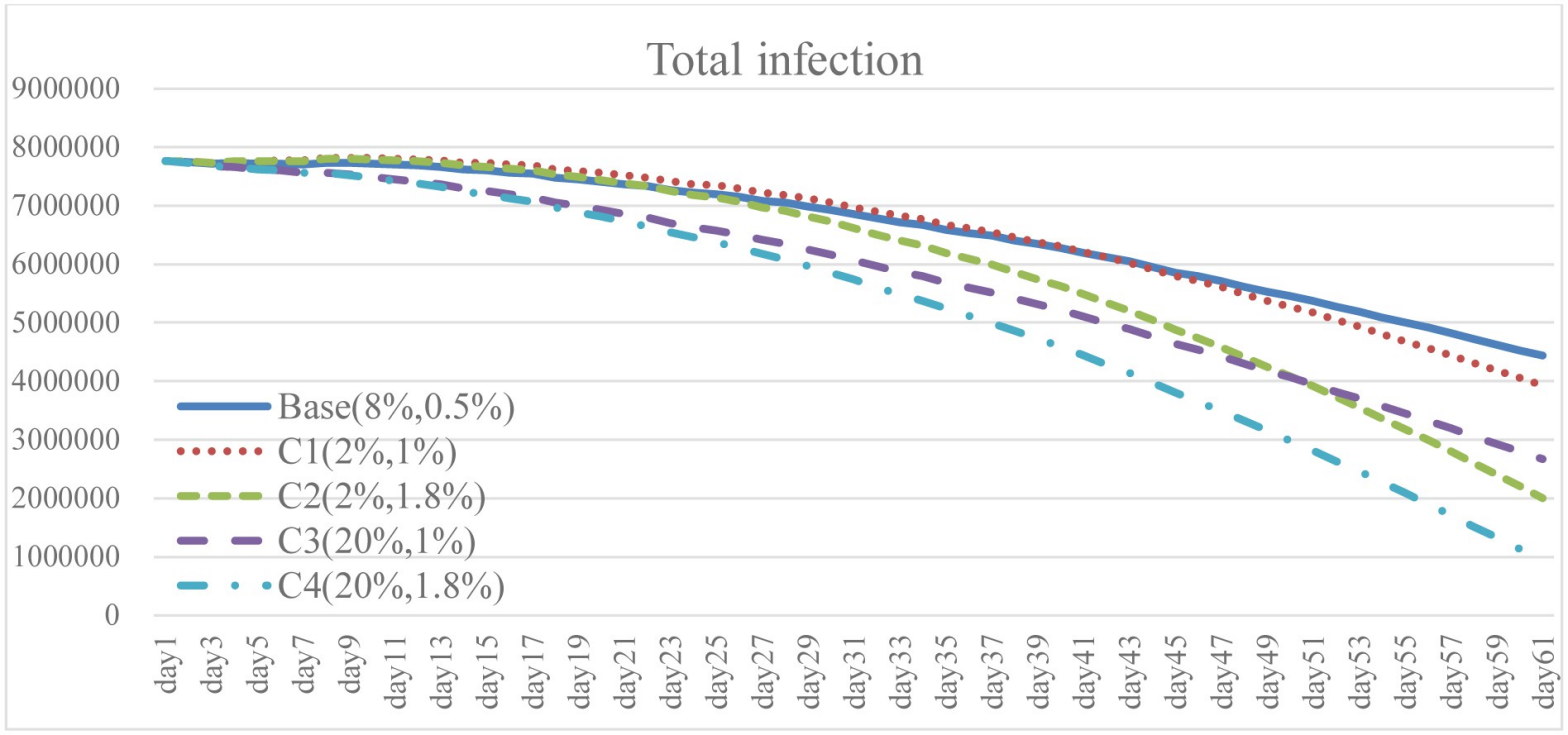
Total infection under different isolation percentage and vaccine supply.

**Fig 17:**
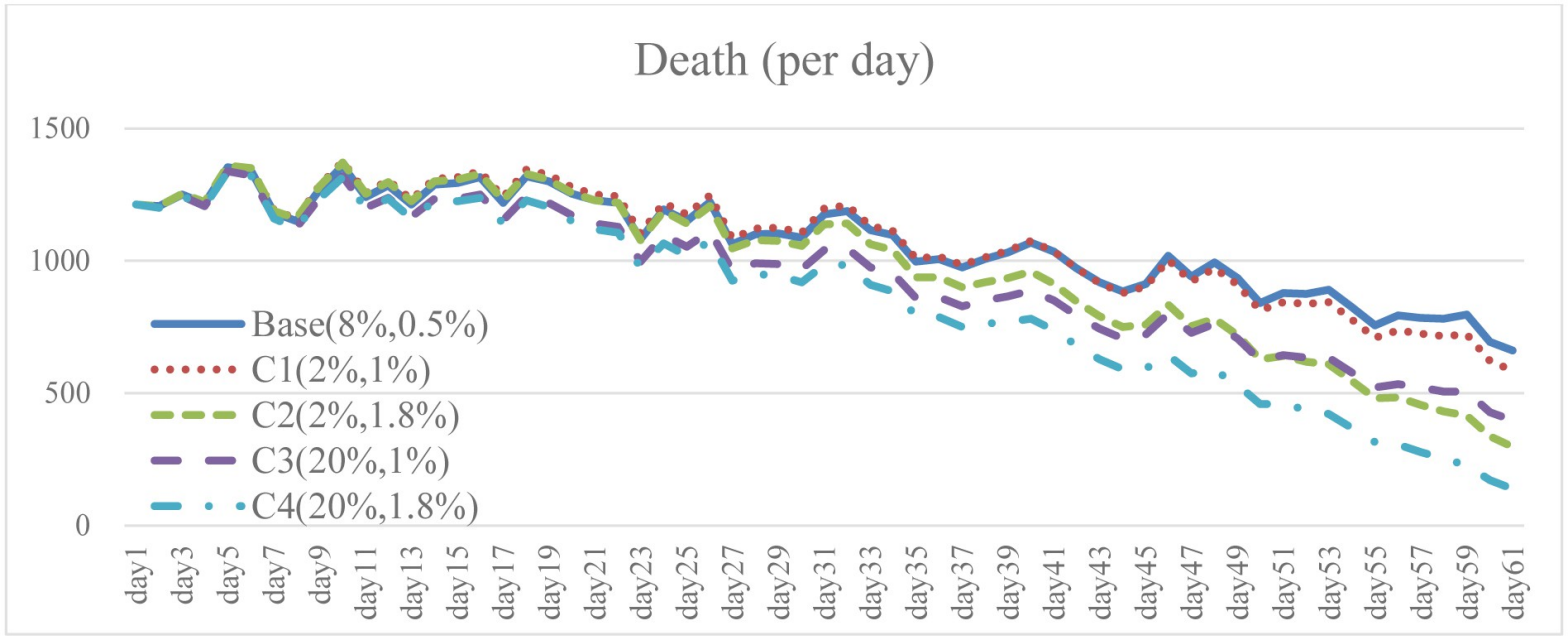
Daily death under different isolation percentage and vaccine supply.

**Fig 18:**
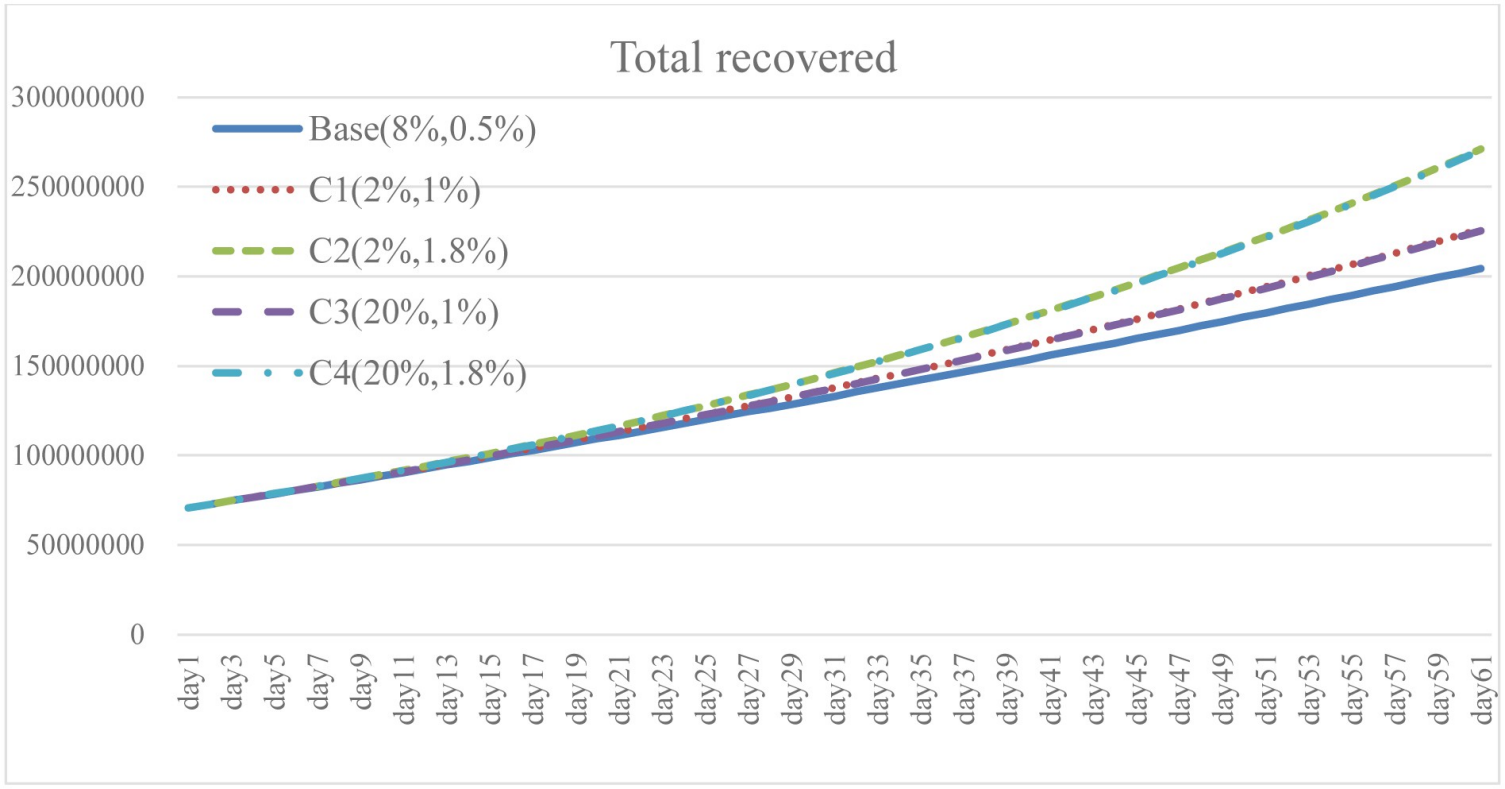
Total recovered under different isolation percentage and vaccine supply.

**Fig 19:**
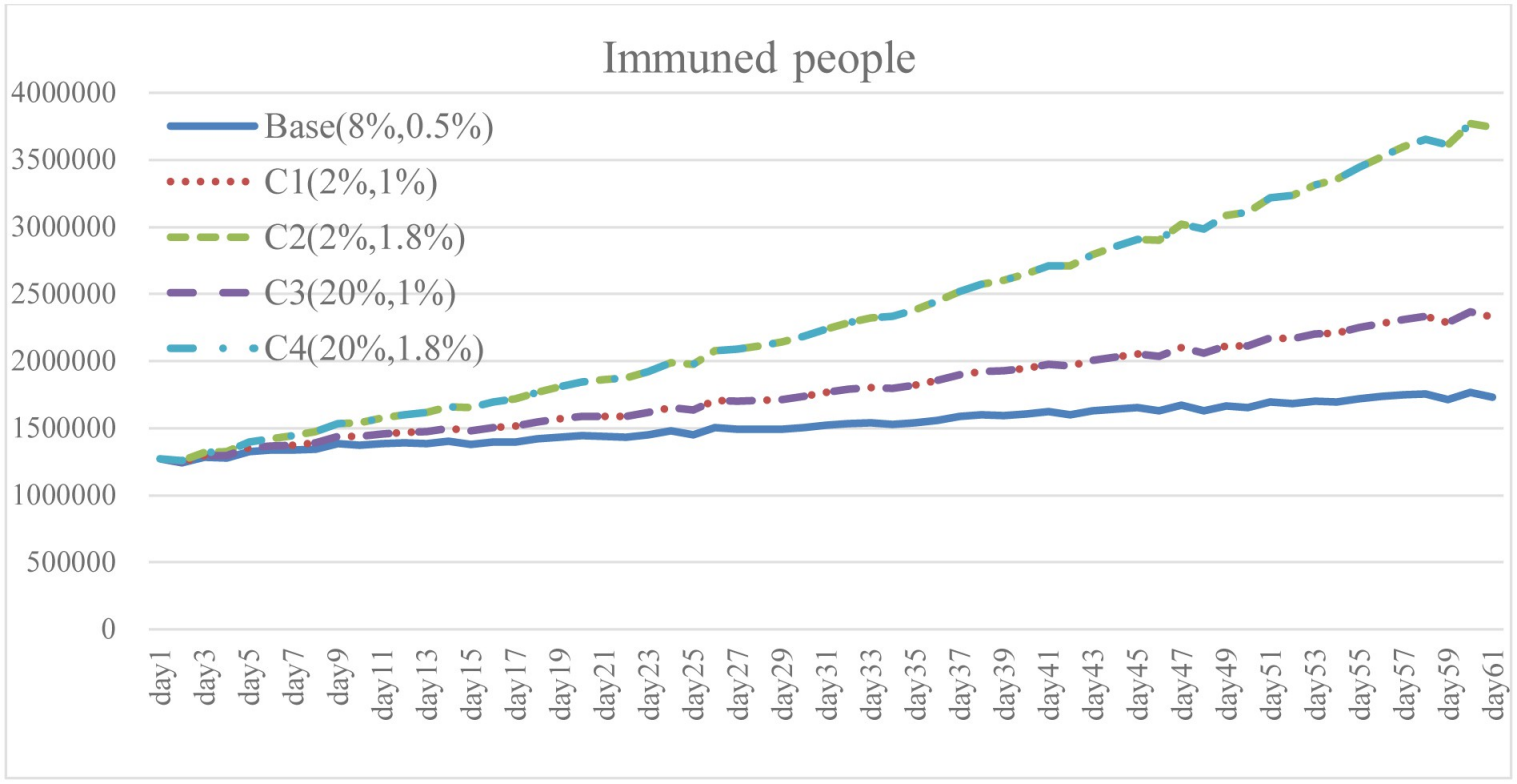
Immuned people under different isolation percentage and vaccine supply.

**Fig 20:**
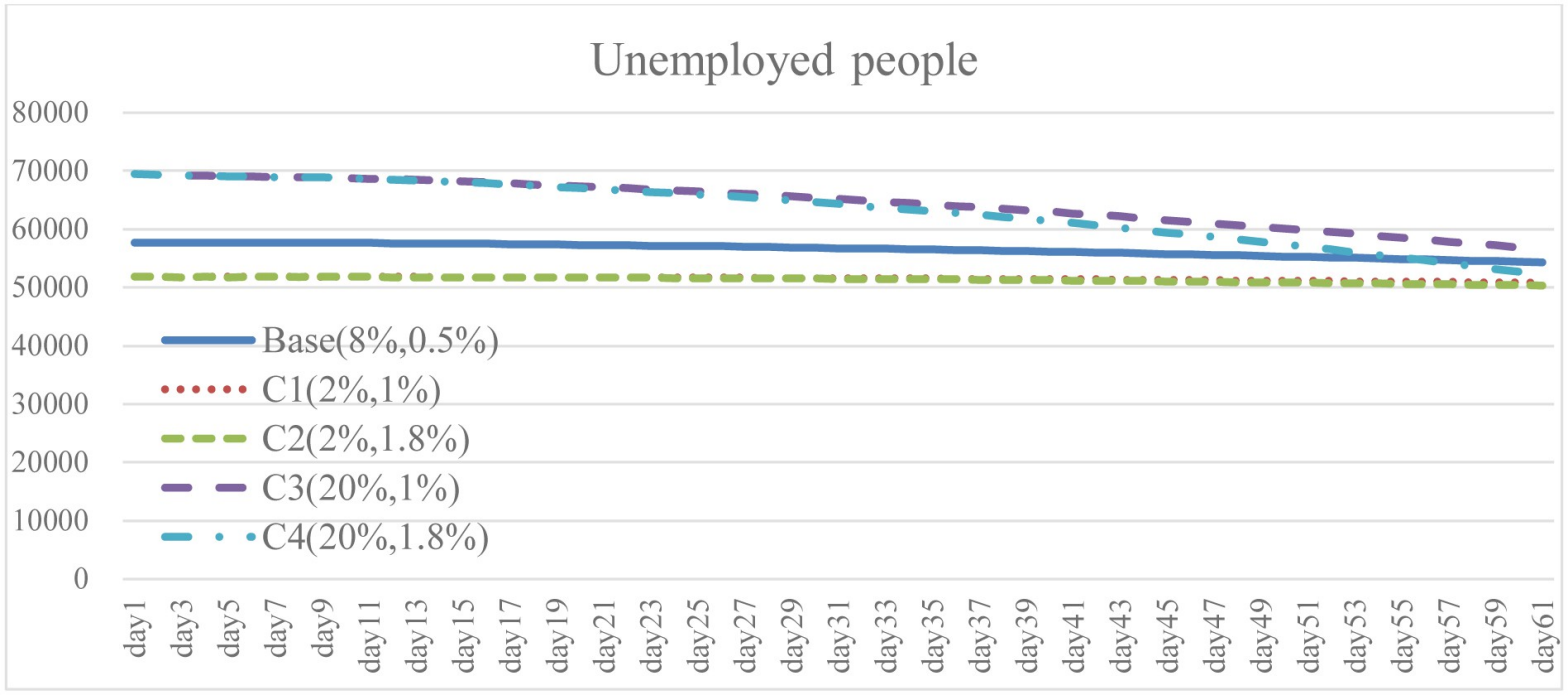
Unemployed people under different isolation percentage and vaccine supply.

**Fig 21:**
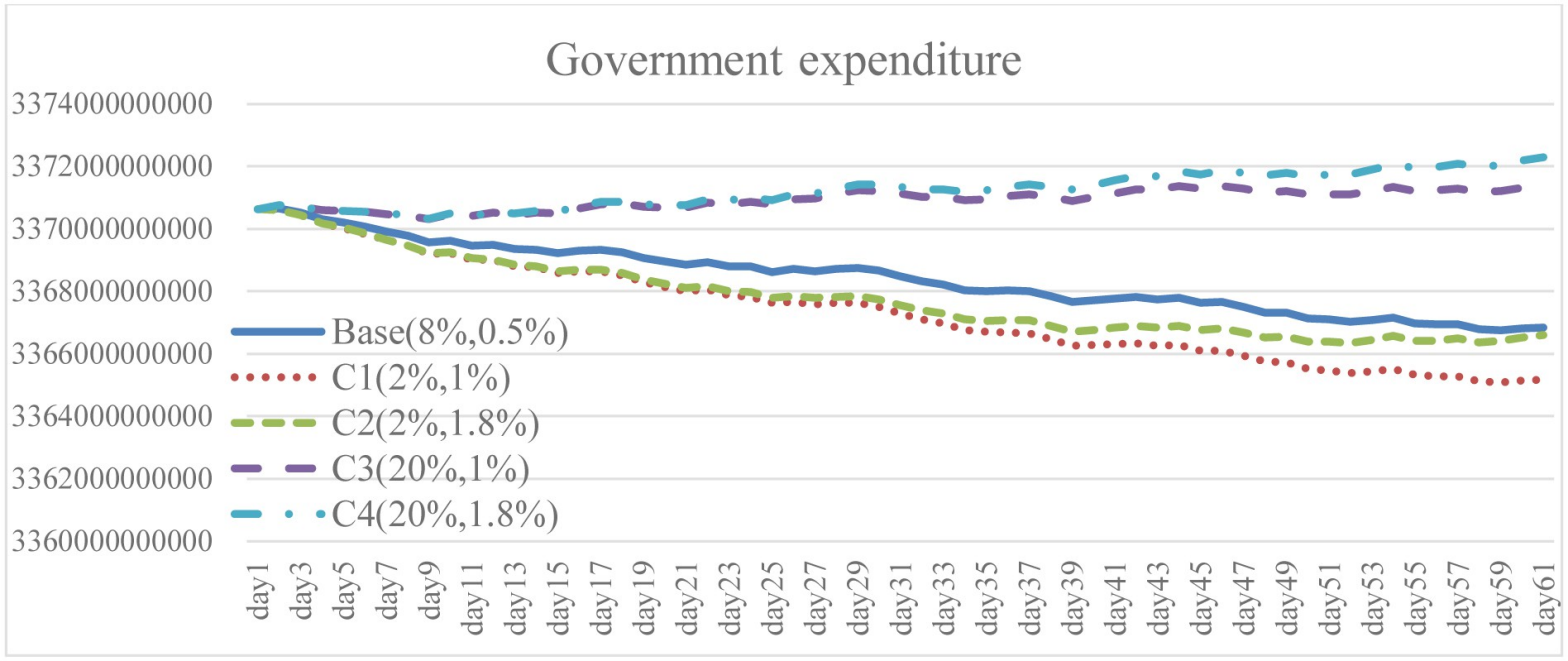
Government expenditure under different isolation percentage and vaccine supply.

To explore the effect of different policy combinations on reducing the spread of COVID-19, this study combined Case A and Case B and derived the combination scenario policy Case C. In the aforementioned simulation results, increasing the isolation percentage and the rate of vaccine supply are both helpful to reduce the spread of COVID-19. From the perspective of combination policy (Figs 16–21), Case C4 is the most effective as a pandemic response, but a large increase in isolation percentage will increase the financial burden of the government. Further, such practice cannot be well improved to mitigate the negative effects on the unemployment situation. Combined with pandemic control and socio-economic development, the simulation results of the Case C2 show better than expected behavior. When the government actively provides vaccine support and people are vaccinated, the total number of infections and deaths on day 60 is significantly reduced by 54.9% compared with the baseline. Even if the isolation rate is reduced, increasing the number of immunized populations is a highly effective pandemic measure. The number of convalesced people and the immunized population significantly increased, 32.6% and 116.2% higher than the baseline, respectively. From the social and economic perspective, the total number of unemployed people is less than that of the baseline and Case C4, and the overall financial burden on the government will not increase. Compared with baseline, it has a trend of decrease. Therefore, for decision makers, implementing an incentive policy to increase vaccination would be a helpful tool to reduce the spread COVID-19. At this time, reducing the isolation percentage is also more conducive to the normal operation of society and the economy.

## 5. Conclusion and discussion

In this paper, we model the impact of isolation and vaccine policies on the progress of the COVID-19 pandemic in the United States. A combination of raising isolation percentage and vaccination rate increase per day can effectively reduce the development of the COVID-19 pandemic. In case C2, the isolation percentage is 2% below the baseline, and vaccine supply increase per day is 1.8%. This is an appropriate combination of policy measures to manage the spread of the pandemic. The simulation results show that the government’s abundant vaccine supply and good vaccination policy with citizen awareness and responsibility will increase the amount of immunized population to 116.2%, which will help reduce the spread of the virus and death. It will not impose a heavy burden on government spending, and unemployment will improve, allowing the United States to steadily return to normal economic activity in the future.

The situations around COVID-19 are dynamic and data can vary significantly. Our model takes into account the current situation in the United States and provides advice based on person-to-person contacts and government efforts to help mitigate the negative effects of the pandemic. This model is likely to be limited to the United States, as vaccines vary in variety and effectiveness in other regions and countries, as well as in government policy patterns and pandemic responses. The frequency of contact between people in isolation is highly uncertain. In the case of vaccination, there are also differences between the willingness of individuals to vaccinate. We used sufficient data to populate the model, but left room to refine the model later. The model makes assumptions about the likely trajectory and socioeconomic impact of COVID-19 through policy measures.

Given the huge challenges posed to public health and society by the emergence of the variants, the process of vaccination should be accelerated. It is also the responsibility of every citizen to get the COVID-19 vaccination. Moreover, there are some people who are unwilling to be vaccinated, either purposively due to distrusting and worrying about side effects or unpurposely such as health restrictions. The government needs to take this into account on how they promote this properly to convince the people. The further modeling also can consider this factor since it gives a systemic effect on spreading the virus. Only after herd immunity is achieved it can be an effective measure in halting the development of the pandemic. Due to the wide-ranging impact of COVID-19, economic development and vaccine distribution across various countries are still topics to be actively discussed in the future. Currently, distribution of vaccines is uneven around the world, so we need to work together to face this global issue.

## Supporting information

S1 Appendix (DOCX)
