# Supplementary material for: System dynamics analysis on the effectiveness of vaccination and social mobilization policies for COVID-19 in the United States
Source: PLoS One. 2022 Aug 12;17(8):e0268443. doi: 10.1371/journal.pone.0268443 (PMC9374237; doi:10.1371/journal.pone.0268443)
Supplement: S1 Appendix — (DOCX) [file pone.0268443.s001.docx]

**Real Data from U.S. within March 1^st^ to March 31^st^**

| **Time** | **Vaccinated population** | **Total death** | **Total infection** |
| --- | --- | --- | --- |
| 01/03/2021 | 50732997 | 504.488 | 7766521 |
| 02/03/2021 | 51755447 | 506216 | 7721619 |
| 03/03/2021 | 52855579 | 508665 | 7676568 |
| 04/03/2021 | 54035670 | 510408 | 7644156 |
| 05/03/2021 | 55547697 | 512629 | 7620254 |
| 06/03/2021 | 57358849 | 514309 | 7578820 |
| 07/03/2021 | 58873710 | 515148 | 7551317 |
| 08/03/2021 | 60005231 | 516044 | 7483742 |
| 09/03/2021 | 61088527 | 517573 | 7439284 |
| 10/03/2021 | 62451150 | 519260 | 7403503 |
| 11/03/2021 | 64071674 | 520885 | 7377820 |
| 12/03/2021 | 65965305 | 522443 | 7341616 |
| 13/03/2021 | 68884011 | 523551 | 7316609 |
| 14/03/2021 | 69784210 | 524257 | 7300740 |
| 15/03/2021 | 71054445 | 525117 | 7246403 |
| 16/03/2021 | 72135616 | 526272 | 7211592 |
| 17/03/2021 | 73669956 | 527660 | 7184632 |
| 18/03/2021 | 75495716 | 529051 | 7169391 |
| 19/03/2021 | 77230061 | 530298 | 7147001 |
| 20/03/2021 | 79367225 | 531119 | 7130172 |
| 21/03/2021 | 81415769 | 531615 | 7108525 |
| 22/03/2021 | 82772416 | 532294 | 7059495 |
| 23/03/2021 | 83930495 | 533321 | 7041293 |
| 24/03/2021 | 85472166 | 534719 | 7017971 |
| 25/03/2021 | 87343622 | 535976 | 7013573 |
| 26/03/2021 | 89559225 | 537274 | 7001454 |
| 27/03/2021 | 91707729 | 538129 | 7002683 |
| 28/03/2021 | 93631163 | 538692 | 6986396 |
| 29/03/2021 | 95015762 | 539381 | 6954196 |
| 30/03/2021 | 96044046 | 540273 | 6939226 |
| 31/03/2021 | 97593290 | 541431 | 6921659 |

**Simulation Data within March 1^st^ to March 31^st^**

| **Time** | **Vaccinated population** | **Total death** | **Total infection** |
| --- | --- | --- | --- |
| 01/03/2021 | 50733000 | 504488 | 7766520 |
| 02/03/2021 | 52007600 | 505700 | 7749020 |
| 03/03/2021 | 53249800 | 506906 | 7717530 |
| 04/03/2021 | 54535000 | 508157 | 7727340 |
| 05/03/2021 | 55812400 | 509375 | 7715320 |
| 06/03/2021 | 57138300 | 510729 | 7718610 |
| 07/03/2021 | 58473000 | 512072 | 7708570 |
| 08/03/2021 | 59808100 | 513253 | 7739130 |
| 09/03/2021 | 61154100 | 514403 | 7733590 |
| 10/03/2021 | 62538100 | 515674 | 7719480 |
| 11/03/2021 | 63911400 | 517034 | 7701500 |
| 12/03/2021 | 65297100 | 518275 | 7685540 |
| 13/03/2021 | 66686300 | 519560 | 7664420 |
| 14/03/2021 | 68073200 | 520774 | 7615640 |
| 15/03/2021 | 69479000 | 522063 | 7600750 |
| 16/03/2021 | 70860400 | 523358 | 7570440 |
| 17/03/2021 | 72258000 | 524675 | 7548240 |
| 18/03/2021 | 73657400 | 525896 | 7480850 |
| 19/03/2021 | 75076300 | 527217 | 7448170 |
| 20/03/2021 | 76511500 | 528517 | 7410550 |
| 21/03/2021 | 77957800 | 529770 | 7367900 |
| 22/03/2021 | 79397400 | 530998 | 7335050 |
| 23/03/2021 | 80829300 | 532218 | 7269230 |
| 24/03/2021 | 82279200 | 533301 | 7223880 |
| 25/03/2021 | 83756900 | 534496 | 7201520 |
| 26/03/2021 | 85208800 | 535648 | 7148670 |
| 27/03/2021 | 86715000 | 536871 | 7087940 |
| 28/03/2021 | 88209100 | 537935 | 7050780 |
| 29/03/2021 | 89702800 | 539035 | 6988140 |
| 30/03/2021 | 91196300 | 540139 | 6928320 |
| 31/03/2021 | 92702300 | 541226 | 6856870 |

**Input Data**

**Social sub-system**

| **Item** | **Parameter** | **Equation** | **Unit** |
| --- | --- | --- | --- |
| 1 | Susceptible | = INTEG (-infection-vaccinated)  Initial value=302274412-50732997=251541415 | person |
| 2 | Total infection | = INTEG (infection-death-recovering)  Initial value= 7766521 | person |
| 3 | Infection | =INTEGER (IF THEN ELSE(transmission rate+ infection for vaccinated-(immune person*vaccinated fraction)<0, 0 , transmission rate+ infection for vaccinated-(immune person*vaccinated fraction) )) | person/day |
| 4 | Total recovered | = INTEG (recovering+ vaccinated)  Initial value=20063666+50732997=70796663 | person |
| 5 | Vaccinated | = INTEGER (immune person) | person/day |
| 6 | Recovering | IF THEN ELSE (Total infection<=0, 0, INTEGER(recovery rate*total infection+ infection for vaccinated) ) | person/day |
| 7 | Total death | = INTEG (death)  Initial value= 504488 | person |
| 8 | Death | = IF THEN ELSE(Total infection<=0, 0 , INTEGER(fatality rate*Total infection)) | person/day |
| 9 | Recovery rate | = RANDOM UNIFORM (0.012, 0.017 , 0 ) | Dmnl |
| 10 | Fatality rate | = 0.00053*RANDOM UNIFORM(0.28, 0.336 , 0) | Dmnl |
| 11 | Isolated people | = isolated percentage*total infection | person |
| 12 | Isolated percentage | = 0.08 | Dmnl |
| 13 | Contact density | = contact rate*(total infection-isolated people) | touch/day |
| 14 | Contact rate | = INTEGER(RANDOM UNIFORM(40, 50, 0 )) | touch/person/day |
| 15 | Transmission rate | = contact density*fraction of susceptible*infectivity | person/day |
| 16 | Infectivity | = RANDOM UNIFORM(0.0007, 0.0008 , 0) | person/day |
| 17 | Fraction of susceptible | = susceptible/total population | Dmnl |
| 18 | Unemployed people | = total population*0.000151+isolated people*0.01265 | person/day |
| 19 | Total population | = total infection+ total recovered+ susceptible | person |

**Economic sub-system**

| **Item** | **Parameter** | **Equation** | **Unit** |
| --- | --- | --- | --- |
| 1 | Government subsidy | = subsidy cost*isolated people+ unemployment benefit* unemployed people | $/day |
| 2 | Unemployment benefit | = 42.86 | $/person/day |
| 3 | Subsidy cost | =100 | $/person/day |
| 4 | Vaccine funding | = J&J vaccine supplies*10+Moderna vaccine supplies*30+Pfizer vaccine supplies*39 | $/day |
| 5 | Government expenditure increment | = changing rate*government expenditure+ vaccine funding+ government subsidy | $ |
| 6 | Changing rate | RANDOM UNIFORM(-0.0000985,-0.0000122 , 0) | Dmnl |
| 7 | Government expenditure | = INTEG(government expenditure increment)  Initial value=3370645000000 | $ |
| 8 | GDP | =INTEG(GDP increment)  Initial value= 19060290000000 | $ |
| 9 | GDP increment | = GDP*growth rate+ government expenditure increment | $ |
| 10 | Growth rate | = RANDOM UNIFORM(0.0001033,0.0001308, 0) | Dmnl |
| 11 | GDP per capita | = GDP/total population | $/person |

**Vaccination subsystem**

| **Item** | **Parameter** | **Equation** | **Unit** |
| --- | --- | --- | --- |
| 1 | Immuned person | = INTEGER(IF THEN ELSE(Susceptible<=0, 0 , "efficacy rate of J&J"*IF THEN ELSE(Time<4, 0 , "J&J vaccine supplies"*doses used)+efficacy rate of Moderna *Moderna vaccine supplies*doses used+efficacy rate of Pfizer*Pfizer vaccine supplies*doses used) ) | person/day |
| 2 | Vaccinated fraction | = 0.05 | Dmnl |
| 3 | Vaccinated population | =INTEGER(vaccination rate)  Initial value= 50732997 | person |
| 4 | Vaccination rate | = immune person | person/ |
| 5 | Vaccinated | = INTEGER(immune person) | person/day |
| 6 | Infection for vaccinated | = J&J vaccine supplies+ Moderna vaccine supplies+ Pfizer vaccine supplies-immune person | person/day |
| 7 | Efficacy rate of Pfizer | = 0.95 | Dmnl |
| 8 | Efficacy rate of Moderna | = 0.94 | Dmnl |
| 9 | Efficacy rate of J&J | = 0.72 | Dmnl |
| 10 | Pfizer vaccine supplies | =INTEG(Pfizer increment)  = 865807 | person |
| 11 | Pfizer increment | =Pfizer vaccine supplies*vaccine increasing | person/day |
| 12 | Moderna vaccine supplies | = INTEG(Moderna increment)  = 865807 | person |
| 13 | Moderna increment | = Moderna vaccine supplies*vaccine increasing | person/day |
| 14 | J&J vaccine supplies | = INTEG(J&J increment)  Initial value= 100000 | person |
| 15 | J&J increment | = "J&J vaccine supplies"*vaccine increasing | person/day |
| 16 | Vaccine increasing | = 0.005 | Dmnl |
| 17 | Doses used | = RANDOM UNIFORM(0.75, 0.78 , 0) |  |

**Experiment Result Data**

**SCENARIO A**

|  | **Total infection** | | |
| --- | --- | --- | --- |
|  | Isolated percentage: 8% | Isolated percentage: 2% | Isolated percentage: 20% |
| day1 | 7766520 | 7766520 | 7766520 |
| day2 | 7749020 | 7760060 | 7726940 |
| day3 | 7717530 | 7738780 | 7675100 |
| day4 | 7727340 | 7760630 | 7661030 |
| day5 | 7715320 | 7759780 | 7626950 |
| day6 | 7718610 | 7774470 | 7607790 |
| day7 | 7708570 | 7777100 | 7572940 |
| day8 | 7739130 | 7821270 | 7576930 |
| day9 | 7733590 | 7827600 | 7548350 |
| day10 | 7719480 | 7824620 | 7512740 |
| day11 | 7701500 | 7818380 | 7472140 |
| day12 | 7685540 | 7813530 | 7434850 |
| day13 | 7664420 | 7804770 | 7390130 |
| day14 | 7615640 | 7766150 | 7322050 |
| day15 | 7600750 | 7763710 | 7283560 |
| day16 | 7570440 | 7744640 | 7232040 |
| day17 | 7548240 | 7734290 | 7187570 |
| day18 | 7480850 | 7675550 | 7104060 |
| day19 | 7448170 | 7653930 | 7050770 |
| day20 | 7410550 | 7626550 | 6994130 |
| day21 | 7367900 | 7593460 | 6933780 |
| day22 | 7335050 | 7571810 | 6880300 |
| day23 | 7269230 | 7514560 | 6798810 |
| day24 | 7223880 | 7478480 | 6736500 |
| day25 | 7201520 | 7467370 | 6693600 |
| day26 | 7148670 | 7423250 | 6624930 |
| day27 | 7087940 | 7370500 | 6549800 |
| day28 | 7050780 | 7343570 | 6494190 |
| day29 | 6988140 | 7290090 | 6415170 |
| day30 | 6928320 | 7239400 | 6339070 |
| day31 | 6856870 | 7175770 | 6253820 |
| day32 | 6780890 | 7107020 | 6165150 |
| day33 | 6719070 | 7053440 | 6088810 |
| day34 | 6676200 | 7019940 | 6029370 |
| day35 | 6594050 | 6944600 | 5935460 |
| day36 | 6538310 | 6898080 | 5863650 |
| day37 | 6485940 | 6854320 | 5796250 |
| day38 | 6410770 | 6785480 | 5710230 |
| day39 | 6346040 | 6728580 | 5632030 |
| day40 | 6283410 | 6674090 | 5555420 |
| day41 | 6199190 | 6596240 | 5460530 |
| day42 | 6128470 | 6532680 | 5377680 |
| day43 | 6050950 | 6461370 | 5289770 |
| day44 | 5952600 | 6367230 | 5184650 |
| day45 | 5858100 | 6277610 | 5082260 |
| day46 | 5794920 | 6221990 | 5006370 |
| day47 | 5719520 | 6153340 | 4919830 |
| day48 | 5618750 | 6056480 | 4813000 |
| day49 | 5528480 | 5970500 | 4715940 |
| day50 | 5456710 | 5905370 | 4633310 |
| day51 | 5374330 | 5828610 | 4541930 |
| day52 | 5276670 | 5734320 | 4439190 |
| day53 | 5188270 | 5650460 | 4343730 |
| day54 | 5095500 | 5561880 | 4244560 |
| day55 | 5003790 | 5474120 | 4146850 |
| day56 | 4914450 | 5388440 | 4051920 |
| day57 | 4820810 | 5298320 | 3953050 |
| day58 | 4718930 | 5198830 | 3847930 |
| day59 | 4630390 | 5114050 | 3753700 |
| day60 | 4528970 | 5014990 | 3649220 |
| day61 | 4440130 | 4929850 | 3554870 |

|  | **Death (per day)** | | |
| --- | --- | --- | --- |
|  | Isolated percentage: 8% | Isolated percentage: 2% | Isolated percentage: 20% |
| day1 | 1212 | 1212 | 1212 |
| day2 | 1206 | 1207 | 1202 |
| day3 | 1251 | 1254 | 1244 |
| day4 | 1218 | 1223 | 1208 |
| day5 | 1354 | 1362 | 1339 |
| day6 | 1343 | 1353 | 1324 |
| day7 | 1181 | 1192 | 1161 |
| day8 | 1150 | 1162 | 1126 |
| day9 | 1271 | 1286 | 1240 |
| day10 | 1360 | 1378 | 1323 |
| day11 | 1241 | 1260 | 1204 |
| day12 | 1285 | 1307 | 1243 |
| day13 | 1214 | 1236 | 1170 |
| day14 | 1289 | 1314 | 1239 |
| day15 | 1295 | 1323 | 1241 |
| day16 | 1317 | 1347 | 1258 |
| day17 | 1221 | 1251 | 1162 |
| day18 | 1321 | 1356 | 1255 |
| day19 | 1300 | 1336 | 1231 |
| day20 | 1253 | 1290 | 1183 |
| day21 | 1228 | 1265 | 1155 |
| day22 | 1220 | 1259 | 1144 |
| day23 | 1083 | 1120 | 1013 |
| day24 | 1195 | 1237 | 1114 |
| day25 | 1152 | 1194 | 1071 |
| day26 | 1223 | 1270 | 1133 |
| day27 | 1064 | 1106 | 983 |
| day28 | 1100 | 1146 | 1013 |
| day29 | 1104 | 1152 | 1013 |
| day30 | 1087 | 1136 | 995 |
| day31 | 1177 | 1232 | 1074 |
| day32 | 1190 | 1247 | 1082 |
| day33 | 1116 | 1172 | 1011 |
| day34 | 1099 | 1156 | 992 |
| day35 | 998 | 1051 | 898 |
| day36 | 1007 | 1062 | 903 |
| day37 | 976 | 1032 | 873 |
| day38 | 1007 | 1066 | 897 |
| day39 | 1033 | 1096 | 917 |
| day40 | 1071 | 1138 | 947 |
| day41 | 1034 | 1100 | 910 |
| day42 | 974 | 1038 | 854 |
| day43 | 920 | 982 | 804 |
| day44 | 885 | 947 | 771 |
| day45 | 913 | 979 | 792 |
| day46 | 1021 | 1096 | 882 |
| day47 | 940 | 1011 | 808 |
| day48 | 996 | 1073 | 853 |
| day49 | 936 | 1010 | 798 |
| day50 | 840 | 909 | 713 |
| day51 | 878 | 952 | 742 |
| day52 | 875 | 951 | 736 |
| day53 | 890 | 969 | 745 |
| day54 | 826 | 901 | 688 |
| day55 | 758 | 830 | 628 |
| day56 | 794 | 870 | 654 |
| day57 | 786 | 864 | 645 |
| day58 | 782 | 862 | 638 |
| day59 | 799 | 883 | 648 |
| day60 | 694 | 769 | 559 |
| day61 | 662 | 735 | 530 |

|  | **Transmission rate** | | |
| --- | --- | --- | --- |
|  | Isolated percentage: 8% | Isolated percentage: 2% | Isolated percentage: 20% |
| day1 | 169263 | 180302 | 147185 |
| day2 | 155858 | 166252 | 135155 |
| day3 | 181194 | 193526 | 156721 |
| day4 | 167432 | 179095 | 144383 |
| day5 | 168366 | 180346 | 144782 |
| day6 | 187231 | 200838 | 160548 |
| day7 | 194686 | 209165 | 166411 |
| day8 | 171181 | 184214 | 145837 |
| day9 | 159403 | 171792 | 135403 |
| day10 | 168561 | 181913 | 142782 |
| day11 | 157013 | 169700 | 132606 |
| day12 | 175986 | 190472 | 148213 |
| day13 | 148890 | 161397 | 124998 |
| day14 | 172755 | 187523 | 144636 |
| day15 | 158380 | 172188 | 132179 |
| day16 | 163455 | 177967 | 136011 |
| day17 | 130428 | 142225 | 108194 |
| day18 | 152855 | 166893 | 126469 |
| day19 | 141222 | 154420 | 116492 |
| day20 | 132151 | 144705 | 108698 |
| day21 | 151762 | 166405 | 124484 |
| day22 | 127242 | 139732 | 104046 |
| day23 | 126835 | 139474 | 103427 |
| day24 | 143017 | 157484 | 116296 |
| day25 | 121363 | 133843 | 98379 |
| day26 | 112608 | 124358 | 91027 |
| day27 | 132285 | 146281 | 106641 |
| day28 | 131420 | 145543 | 105615 |
| day29 | 128820 | 142882 | 103200 |
| day30 | 115860 | 128703 | 92525 |
| day31 | 109394 | 121696 | 87099 |
| day32 | 114080 | 127091 | 90560 |
| day33 | 116504 | 129986 | 92196 |
| day34 | 107777 | 120434 | 85014 |
| day35 | 122670 | 137280 | 96459 |
| day36 | 109349 | 122576 | 85685 |
| day37 | 92321 | 103650 | 72102 |
| day38 | 105207 | 118291 | 81910 |
| day39 | 107916 | 121534 | 83731 |
| day40 | 100360 | 113212 | 77591 |
| day41 | 98600 | 111410 | 75962 |
| day42 | 91745 | 103838 | 70427 |
| day43 | 81462 | 92350 | 62312 |
| day44 | 87345 | 99178 | 66580 |
| day45 | 93769 | 106654 | 71212 |
| day46 | 95971 | 109356 | 72596 |
| day47 | 79199 | 90413 | 59665 |
| day48 | 75679 | 86548 | 56788 |
| day49 | 86801 | 99442 | 64878 |
| day50 | 85165 | 97759 | 63379 |
| day51 | 68121 | 78348 | 50471 |
| day52 | 74854 | 86255 | 55222 |
| day53 | 74526 | 86049 | 54730 |
| day54 | 69927 | 80907 | 51109 |
| day55 | 60670 | 70343 | 44129 |
| day56 | 63793 | 74118 | 46176 |
| day57 | 58196 | 67764 | 41908 |
| day58 | 58620 | 68410 | 41991 |
| day59 | 61484 | 71918 | 43799 |
| day60 | 57565 | 67495 | 40773 |
| day61 | 54037 | 63516 | 38044 |

|  | **Unemployed people** | | |
| --- | --- | --- | --- |
|  | Isolated percentage: 8% | Isolated percentage: 2% | Isolated percentage: 20% |
| day1 | 57706 | 51811 | 69495 |
| day2 | 57688 | 51809 | 69395 |
| day3 | 57656 | 51803 | 69263 |
| day4 | 57665 | 51809 | 69228 |
| day5 | 57653 | 51808 | 69141 |
| day6 | 57656 | 51812 | 69093 |
| day7 | 57646 | 51812 | 69004 |
| day8 | 57676 | 51823 | 69014 |
| day9 | 57671 | 51825 | 68942 |
| day10 | 57656 | 51824 | 68851 |
| day11 | 57638 | 51822 | 68748 |
| day12 | 57621 | 51821 | 68654 |
| day13 | 57600 | 51818 | 68541 |
| day14 | 57550 | 51808 | 68368 |
| day15 | 57535 | 51807 | 68271 |
| day16 | 57504 | 51802 | 68140 |
| day17 | 57482 | 51799 | 68027 |
| day18 | 57413 | 51784 | 67816 |
| day19 | 57380 | 51779 | 67681 |
| day20 | 57342 | 51772 | 67537 |
| day21 | 57298 | 51763 | 67385 |
| day22 | 57265 | 51757 | 67249 |
| day23 | 57198 | 51743 | 67043 |
| day24 | 57152 | 51733 | 66885 |
| day25 | 57129 | 51730 | 66776 |
| day26 | 57076 | 51719 | 66602 |
| day27 | 57014 | 51706 | 66412 |
| day28 | 56976 | 51699 | 66271 |
| day29 | 56913 | 51685 | 66071 |
| day30 | 56852 | 51672 | 65878 |
| day31 | 56779 | 51656 | 65663 |
| day32 | 56702 | 51638 | 65438 |
| day33 | 56640 | 51624 | 65245 |
| day34 | 56596 | 51616 | 65094 |
| day35 | 56513 | 51596 | 64857 |
| day36 | 56456 | 51584 | 64675 |
| day37 | 56403 | 51573 | 64504 |
| day38 | 56327 | 51556 | 64286 |
| day39 | 56261 | 51541 | 64088 |
| day40 | 56198 | 51527 | 63894 |
| day41 | 56112 | 51507 | 63654 |
| day42 | 56040 | 51491 | 63444 |
| day43 | 55962 | 51473 | 63222 |
| day44 | 55862 | 51449 | 62956 |
| day45 | 55766 | 51426 | 62697 |
| day46 | 55702 | 51412 | 62505 |
| day47 | 55626 | 51394 | 62285 |
| day48 | 55524 | 51370 | 62015 |
| day49 | 55432 | 51348 | 61769 |
| day50 | 55360 | 51331 | 61560 |
| day51 | 55276 | 51312 | 61329 |
| day52 | 55177 | 51288 | 61069 |
| day53 | 55087 | 51266 | 60827 |
| day54 | 54993 | 51244 | 60576 |
| day55 | 54901 | 51221 | 60329 |
| day56 | 54810 | 51199 | 60089 |
| day57 | 54715 | 51177 | 59838 |
| day58 | 54612 | 51151 | 59572 |
| day59 | 54522 | 51130 | 59334 |
| day60 | 54419 | 51104 | 59069 |
| day61 | 54329 | 51083 | 58831 |

|  | **Government expenditure** | | |
| --- | --- | --- | --- |
|  | Isolated percentage: 8% | Isolated percentage: 2% | Isolated percentage: 20% |
| day1 | 3370640000000 | 3370640000000 | 3370640000000 |
| day2 | 3370660000000 | 3370660000000 | 3370760000000 |
| day3 | 3370530000000 | 3370630000000 | 3370720000000 |
| day4 | 3370320000000 | 3370470000000 | 3370600000000 |
| day5 | 3370220000000 | 3370520000000 | 3370590000000 |
| day6 | 3370080000000 | 3370420000000 | 3370550000000 |
| day7 | 3369920000000 | 3370470000000 | 3370470000000 |
| day8 | 3369780000000 | 3370530000000 | 3370420000000 |
| day9 | 3369580000000 | 3370360000000 | 3370310000000 |
| day10 | 3369640000000 | 3370350000000 | 3370460000000 |
| day11 | 3369480000000 | 3370230000000 | 3370390000000 |
| day12 | 3369500000000 | 3370210000000 | 3370500000000 |
| day13 | 3369350000000 | 3370130000000 | 3370440000000 |
| day14 | 3369340000000 | 3370130000000 | 3370520000000 |
| day15 | 3369220000000 | 3370200000000 | 3370480000000 |
| day16 | 3369300000000 | 3370210000000 | 3370640000000 |
| day17 | 3369340000000 | 3370060000000 | 3370770000000 |
| day18 | 3369260000000 | 3370050000000 | 3370770000000 |
| day19 | 3369080000000 | 3369920000000 | 3370680000000 |
| day20 | 3368970000000 | 3369990000000 | 3370650000000 |
| day21 | 3368860000000 | 3369910000000 | 3370620000000 |
| day22 | 3368950000000 | 3369920000000 | 3370790000000 |
| day23 | 3368820000000 | 3369960000000 | 3370740000000 |
| day24 | 3368800000000 | 3369790000000 | 3370800000000 |
| day25 | 3368630000000 | 3369650000000 | 3370710000000 |
| day26 | 3368720000000 | 3369560000000 | 3370870000000 |
| day27 | 3368660000000 | 3369530000000 | 3370890000000 |
| day28 | 3368730000000 | 3369410000000 | 3371030000000 |
| day29 | 3368760000000 | 3369410000000 | 3371130000000 |
| day30 | 3368670000000 | 3369490000000 | 3371120000000 |
| day31 | 3368500000000 | 3369370000000 | 3371020000000 |
| day32 | 3368320000000 | 3369210000000 | 3370910000000 |
| day33 | 3368230000000 | 3369050000000 | 3370890000000 |
| day34 | 3368050000000 | 3368940000000 | 3370770000000 |
| day35 | 3368020000000 | 3368750000000 | 3370810000000 |
| day36 | 3368030000000 | 3368650000000 | 3370890000000 |
| day37 | 3368020000000 | 3368590000000 | 3370950000000 |
| day38 | 3367860000000 | 3368390000000 | 3370850000000 |
| day39 | 3367660000000 | 3368360000000 | 3370720000000 |
| day40 | 3367720000000 | 3368260000000 | 3370830000000 |
| day41 | 3367780000000 | 3368330000000 | 3370950000000 |
| day42 | 3367830000000 | 3368190000000 | 3371060000000 |
| day43 | 3367750000000 | 3368060000000 | 3371050000000 |
| day44 | 3367800000000 | 3367870000000 | 3371150000000 |
| day45 | 3367640000000 | 3367820000000 | 3371050000000 |
| day46 | 3367660000000 | 3367790000000 | 3371120000000 |
| day47 | 3367510000000 | 3367740000000 | 3371030000000 |
| day48 | 3367330000000 | 3367540000000 | 3370900000000 |
| day49 | 3367320000000 | 3367530000000 | 3370940000000 |
| day50 | 3367140000000 | 3367350000000 | 3370810000000 |
| day51 | 3367100000000 | 3367330000000 | 3370820000000 |
| day52 | 3367020000000 | 3367340000000 | 3370790000000 |
| day53 | 3367090000000 | 3367380000000 | 3370910000000 |
| day54 | 3367170000000 | 3367390000000 | 3371030000000 |
| day55 | 3366980000000 | 3367280000000 | 3370890000000 |
| day56 | 3366940000000 | 3367300000000 | 3370890000000 |
| day57 | 3366950000000 | 3367300000000 | 3370940000000 |
| day58 | 3366790000000 | 3367340000000 | 3370820000000 |
| day59 | 3366760000000 | 3367150000000 | 3370830000000 |
| day60 | 3366820000000 | 3367060000000 | 3370930000000 |
| day61 | 3366860000000 | 3366880000000 | 3371000000000 |

**SCENARIO B**

|  | **Total infection** | | |
| --- | --- | --- | --- |
|  | Vaccine supplies increasing per day: 0.5% | Vaccine supplies increasing per day: 1% | Vaccine supplies increasing per day: 1.8% |
| day1 | 7766520 | 7766520 | 7766520 |
| day2 | 7749020 | 7749020 | 7749020 |
| day3 | 7717530 | 7717220 | 7716720 |
| day4 | 7727340 | 7726380 | 7724830 |
| day5 | 7715320 | 7713380 | 7710240 |
| day6 | 7718610 | 7715280 | 7709890 |
| day7 | 7708570 | 7703470 | 7695180 |
| day8 | 7739130 | 7731830 | 7719920 |
| day9 | 7733590 | 7723720 | 7707540 |
| day10 | 7719480 | 7706570 | 7685300 |
| day11 | 7701500 | 7685140 | 7658050 |
| day12 | 7685540 | 7665250 | 7631550 |
| day13 | 7664420 | 7639720 | 7598520 |
| day14 | 7615640 | 7586220 | 7536940 |
| day15 | 7600750 | 7565880 | 7507220 |
| day16 | 7570440 | 7529830 | 7461230 |
| day17 | 7548240 | 7501250 | 7421560 |
| day18 | 7480850 | 7427400 | 7336370 |
| day19 | 7448170 | 7387300 | 7283230 |
| day20 | 7410550 | 7341750 | 7223640 |
| day21 | 7367900 | 7290680 | 7157560 |
| day22 | 7335050 | 7248620 | 7099030 |
| day23 | 7269230 | 7173650 | 7007580 |
| day24 | 7223880 | 7118220 | 6933930 |
| day25 | 7201520 | 7084480 | 6879550 |
| day26 | 7148670 | 7020540 | 6795330 |
| day27 | 7087940 | 6947980 | 6701000 |
| day28 | 7050780 | 6897710 | 6626550 |
| day29 | 6988140 | 6821820 | 6526100 |
| day30 | 6928320 | 6748070 | 6426440 |
| day31 | 6856870 | 6662490 | 6314340 |
| day32 | 6780890 | 6571840 | 6196010 |
| day33 | 6719070 | 6494030 | 6088000 |
| day34 | 6676200 | 6433730 | 5994710 |
| day35 | 6594050 | 6335170 | 5864780 |
| day36 | 6538310 | 6260730 | 5754710 |
| day37 | 6485940 | 6188890 | 5645580 |
| day38 | 6410770 | 6094770 | 5514700 |
| day39 | 6346040 | 6009190 | 5388810 |
| day40 | 6283410 | 5924740 | 5262110 |
| day41 | 6199190 | 5819300 | 5115250 |
| day42 | 6128470 | 5725600 | 4976640 |
| day43 | 6050950 | 5625350 | 4831730 |
| day44 | 5952600 | 5505100 | 4667960 |
| day45 | 5858100 | 5387180 | 4503600 |
| day46 | 5794920 | 5296760 | 4359590 |
| day47 | 5719520 | 5194490 | 4204440 |
| day48 | 5618750 | 5068610 | 4028270 |
| day49 | 5528480 | 4952480 | 3860370 |
| day50 | 5456710 | 4850680 | 3699130 |
| day51 | 5374330 | 4738810 | 3528880 |
| day52 | 5276670 | 4613240 | 3346860 |
| day53 | 5188270 | 4494670 | 3168040 |
| day54 | 5095500 | 4371100 | 2983000 |
| day55 | 5003790 | 4248100 | 2797410 |
| day56 | 4914450 | 4126660 | 2611010 |
| day57 | 4820810 | 4000080 | 2418270 |
| day58 | 4718930 | 3866090 | 2219240 |
| day59 | 4630390 | 3742110 | 2024090 |
| day60 | 4528970 | 3607630 | 1824210 |
| day61 | 4440130 | 3481500 | 1624000 |

|  | **Death (per day)** | | |
| --- | --- | --- | --- |
|  | Vaccine supplies increasing per day: 0.5% | Vaccine supplies increasing per day: 1% | Vaccine supplies increasing per day: 1.8% |
| day1 | 1212 | 1212 | 1212 |
| day2 | 1206 | 1206 | 1206 |
| day3 | 1251 | 1251 | 1251 |
| day4 | 1218 | 1218 | 1218 |
| day5 | 1354 | 1354 | 1354 |
| day6 | 1343 | 1343 | 1342 |
| day7 | 1181 | 1181 | 1179 |
| day8 | 1150 | 1149 | 1147 |
| day9 | 1271 | 1269 | 1266 |
| day10 | 1360 | 1357 | 1353 |
| day11 | 1241 | 1239 | 1234 |
| day12 | 1285 | 1282 | 1276 |
| day13 | 1214 | 1210 | 1203 |
| day14 | 1289 | 1284 | 1275 |
| day15 | 1295 | 1289 | 1279 |
| day16 | 1317 | 1310 | 1298 |
| day17 | 1221 | 1213 | 1200 |
| day18 | 1321 | 1312 | 1296 |
| day19 | 1300 | 1289 | 1271 |
| day20 | 1253 | 1242 | 1222 |
| day21 | 1228 | 1215 | 1193 |
| day22 | 1220 | 1205 | 1180 |
| day23 | 1083 | 1069 | 1044 |
| day24 | 1195 | 1177 | 1147 |
| day25 | 1152 | 1133 | 1100 |
| day26 | 1223 | 1201 | 1163 |
| day27 | 1064 | 1043 | 1005 |
| day28 | 1100 | 1076 | 1034 |
| day29 | 1104 | 1078 | 1031 |
| day30 | 1087 | 1059 | 1009 |
| day31 | 1177 | 1144 | 1084 |
| day32 | 1190 | 1153 | 1087 |
| day33 | 1116 | 1079 | 1011 |
| day34 | 1099 | 1059 | 987 |
| day35 | 998 | 959 | 887 |
| day36 | 1007 | 964 | 886 |
| day37 | 976 | 932 | 850 |
| day38 | 1007 | 957 | 866 |
| day39 | 1033 | 979 | 877 |
| day40 | 1071 | 1010 | 897 |
| day41 | 1034 | 970 | 853 |
| day42 | 974 | 910 | 791 |
| day43 | 920 | 855 | 734 |
| day44 | 885 | 818 | 694 |
| day45 | 913 | 840 | 702 |
| day46 | 1021 | 933 | 768 |
| day47 | 940 | 853 | 691 |
| day48 | 996 | 898 | 714 |
| day49 | 936 | 838 | 653 |
| day50 | 840 | 747 | 569 |
| day51 | 878 | 774 | 576 |
| day52 | 875 | 765 | 555 |
| day53 | 890 | 771 | 543 |
| day54 | 826 | 708 | 483 |
| day55 | 758 | 644 | 424 |
| day56 | 794 | 666 | 421 |
| day57 | 786 | 652 | 394 |
| day58 | 782 | 641 | 368 |
| day59 | 799 | 646 | 349 |
| day60 | 694 | 553 | 279 |
| day61 | 662 | 519 | 242 |

|  | **Immuned people** | | |
| --- | --- | --- | --- |
|  | Vaccine supplies increasing per day: 0.5% | Vaccine supplies increasing per day: 1% | Vaccine supplies increasing per day: 1.8% |
| day1 | 1274610 | 1274610 | 1274610 |
| day2 | 1242230 | 1248410 | 1258300 |
| day3 | 1285190 | 1298000 | 1318650 |
| day4 | 1277410 | 1296570 | 1327630 |
| day5 | 1325910 | 1352490 | 1395860 |
| day6 | 1334630 | 1368160 | 1423210 |
| day7 | 1335170 | 1375520 | 1442200 |
| day8 | 1345980 | 1393560 | 1472680 |
| day9 | 1383970 | 1440030 | 1533850 |
| day10 | 1373300 | 1436030 | 1541700 |
| day11 | 1385730 | 1456240 | 1575780 |
| day12 | 1389140 | 1467080 | 1600090 |
| day13 | 1386930 | 1472030 | 1618210 |
| day14 | 1405840 | 1499530 | 1661490 |
| day15 | 1381370 | 1480760 | 1653690 |
| day16 | 1397650 | 1505660 | 1694820 |
| day17 | 1399330 | 1514980 | 1718810 |
| day18 | 1418940 | 1543850 | 1765440 |
| day19 | 1435180 | 1569280 | 1808740 |
| day20 | 1446320 | 1589330 | 1846360 |
| day21 | 1439610 | 1589830 | 1861560 |
| day22 | 1431940 | 1589230 | 1875600 |
| day23 | 1449840 | 1617100 | 1923620 |
| day24 | 1477740 | 1656420 | 1985990 |
| day25 | 1451870 | 1635520 | 1976470 |
| day26 | 1506190 | 1705150 | 2076930 |
| day27 | 1494130 | 1699920 | 2086960 |
| day28 | 1493660 | 1707830 | 2113290 |
| day29 | 1493480 | 1716120 | 2140360 |
| day30 | 1506040 | 1739160 | 2186280 |
| day31 | 1523050 | 1767550 | 2239570 |
| day32 | 1534500 | 1789710 | 2285600 |
| day33 | 1538370 | 1803150 | 2321010 |
| day34 | 1526490 | 1798120 | 2332860 |
| day35 | 1538280 | 1821030 | 2381310 |
| day36 | 1559820 | 1855710 | 2445870 |
| day37 | 1587330 | 1897830 | 2521200 |
| day38 | 1600550 | 1923160 | 2575090 |
| day39 | 1596090 | 1927340 | 2601130 |
| day40 | 1604340 | 1946940 | 2648400 |
| day41 | 1622790 | 1979130 | 2713500 |
| day42 | 1601380 | 1962740 | 2712350 |
| day43 | 1627030 | 2004090 | 2791430 |
| day44 | 1641020 | 2031380 | 2851860 |
| day45 | 1652120 | 2055300 | 2908280 |
| day46 | 1628120 | 2035530 | 2903120 |
| day47 | 1672780 | 2101760 | 3021330 |
| day48 | 1630680 | 2059050 | 2983380 |
| day49 | 1666890 | 2115250 | 3089080 |
| day50 | 1655490 | 2111240 | 3107640 |
| day51 | 1693330 | 2170240 | 3219790 |
| day52 | 1681210 | 2165430 | 3238100 |
| day53 | 1699850 | 2200320 | 3316340 |
| day54 | 1697260 | 2207900 | 3354130 |
| day55 | 1721940 | 2251150 | 3446920 |
| day56 | 1736920 | 2282030 | 3521870 |
| day57 | 1751830 | 2313080 | 3598060 |
| day58 | 1757500 | 2332100 | 3656390 |
| day59 | 1713150 | 2284560 | 3610230 |
| day60 | 1766680 | 2367660 | 3771190 |
| day61 | 1730400 | 2330590 | 3741540 |

|  | **Total recovered** | | |
| --- | --- | --- | --- |
|  | Vaccine supplies increasing per day: 0.5% | Vaccine supplies increasing per day: 1% | Vaccine supplies increasing per day: 1.8% |
| day1 | 70796700 | 70796700 | 70796700 |
| day2 | 72750100 | 72750100 | 72750100 |
| day3 | 74714900 | 74724100 | 74738800 |
| day4 | 76670800 | 76698400 | 76742800 |
| day5 | 78644400 | 78699900 | 78789400 |
| day6 | 80610300 | 80703200 | 80853900 |
| day7 | 82617400 | 82757400 | 82985400 |
| day8 | 84600800 | 84797800 | 85120000 |
| day9 | 86605800 | 86869800 | 87303200 |
| day10 | 88615000 | 88956000 | 89518500 |
| day11 | 90647200 | 91075600 | 91785100 |
| day12 | 92675000 | 93201000 | 94076300 |
| day13 | 94736200 | 95370500 | 96430500 |
| day14 | 96807900 | 97561200 | 98825400 |
| day15 | 98878300 | 99761300 | 101250000 |
| day16 | 100961000 | 101984000 | 103718000 |
| day17 | 103049000 | 104225000 | 106225000 |
| day18 | 105159000 | 106498000 | 108786000 |
| day19 | 107266000 | 108780000 | 111377000 |
| day20 | 109376000 | 111076000 | 114006000 |
| day21 | 111491000 | 113389000 | 116675000 |
| day22 | 113626000 | 115734000 | 119400000 |
| day23 | 115780000 | 118110000 | 122181000 |
| day24 | 117923000 | 120487000 | 124988000 |
| day25 | 120067000 | 122879000 | 127835000 |
| day26 | 122232000 | 125303000 | 130742000 |
| day27 | 124404000 | 127747000 | 133695000 |
| day28 | 126583000 | 130212000 | 136696000 |
| day29 | 128797000 | 132723000 | 139772000 |
| day30 | 131016000 | 135254000 | 142896000 |
| day31 | 133243000 | 137806000 | 146071000 |
| day32 | 135478000 | 140380000 | 149299000 |
| day33 | 137714000 | 142968000 | 152573000 |
| day34 | 139944000 | 145565000 | 155888000 |
| day35 | 142216000 | 148217000 | 159289000 |
| day36 | 144487000 | 150883000 | 162738000 |
| day37 | 146750000 | 153556000 | 166231000 |
| day38 | 149029000 | 156260000 | 169788000 |
| day39 | 151321000 | 158992000 | 173408000 |
| day40 | 153625000 | 161750000 | 177092000 |
| day41 | 155953000 | 164547000 | 180852000 |
| day42 | 158276000 | 167356000 | 184662000 |
| day43 | 160611000 | 170193000 | 188540000 |
| day44 | 162967000 | 173065000 | 192493000 |
| day45 | 165336000 | 175966000 | 196515000 |
| day46 | 167690000 | 178872000 | 200587000 |
| day47 | 170072000 | 181819000 | 204742000 |
| day48 | 172471000 | 184801000 | 208973000 |
| day49 | 174870000 | 187800000 | 213269000 |
| day50 | 177271000 | 190819000 | 217633000 |
| day51 | 179694000 | 193876000 | 222080000 |
| day52 | 182125000 | 196959000 | 226602000 |
| day53 | 184565000 | 200070000 | 231201000 |
| day54 | 187020000 | 203214000 | 235883000 |
| day55 | 189482000 | 206382000 | 240642000 |
| day56 | 191943000 | 209571000 | 245477000 |
| day57 | 194423000 | 212795000 | 250400000 |
| day58 | 196916000 | 216052000 | 255410000 |
| day59 | 199408000 | 219330000 | 260501000 |
| day60 | 201931000 | 222654000 | 265689000 |
| day61 | 204447000 | 225994000 | 270960000 |

|  | **Unemployed people** | | |
| --- | --- | --- | --- |
|  | Vaccine supplies increasing per day: 0.5% | Vaccine supplies increasing per day: 1% | Vaccine supplies increasing per day: 1.8% |
| day1 | 57706 | 57706 | 57706 |
| day2 | 57688 | 57688 | 57688 |
| day3 | 57656 | 57655 | 57655 |
| day4 | 57665 | 57664 | 57663 |
| day5 | 57653 | 57651 | 57648 |
| day6 | 57656 | 57653 | 57647 |
| day7 | 57646 | 57641 | 57632 |
| day8 | 57676 | 57669 | 57657 |
| day9 | 57671 | 57661 | 57644 |
| day10 | 57656 | 57643 | 57622 |
| day11 | 57638 | 57621 | 57594 |
| day12 | 57621 | 57601 | 57567 |
| day13 | 57600 | 57575 | 57533 |
| day14 | 57550 | 57521 | 57471 |
| day15 | 57535 | 57500 | 57440 |
| day16 | 57504 | 57463 | 57394 |
| day17 | 57482 | 57434 | 57353 |
| day18 | 57413 | 57359 | 57267 |
| day19 | 57380 | 57318 | 57213 |
| day20 | 57342 | 57272 | 57153 |
| day21 | 57298 | 57220 | 57085 |
| day22 | 57265 | 57177 | 57026 |
| day23 | 57198 | 57101 | 56933 |
| day24 | 57152 | 57045 | 56859 |
| day25 | 57129 | 57011 | 56803 |
| day26 | 57076 | 56946 | 56718 |
| day27 | 57014 | 56872 | 56622 |
| day28 | 56976 | 56821 | 56547 |
| day29 | 56913 | 56744 | 56445 |
| day30 | 56852 | 56669 | 56344 |
| day31 | 56779 | 56583 | 56230 |
| day32 | 56702 | 56491 | 56111 |
| day33 | 56640 | 56412 | 56001 |
| day34 | 56596 | 56351 | 55907 |
| day35 | 56513 | 56251 | 55775 |
| day36 | 56456 | 56175 | 55663 |
| day37 | 56403 | 56102 | 55553 |
| day38 | 56327 | 56007 | 55420 |
| day39 | 56261 | 55920 | 55293 |
| day40 | 56198 | 55835 | 55164 |
| day41 | 56112 | 55728 | 55016 |
| day42 | 56040 | 55633 | 54875 |
| day43 | 55962 | 55531 | 54728 |
| day44 | 55862 | 55409 | 54563 |
| day45 | 55766 | 55290 | 54396 |
| day46 | 55702 | 55198 | 54250 |
| day47 | 55626 | 55095 | 54093 |
| day48 | 55524 | 54967 | 53915 |
| day49 | 55432 | 54850 | 53745 |
| day50 | 55360 | 54746 | 53581 |
| day51 | 55276 | 54633 | 53409 |
| day52 | 55177 | 54506 | 53225 |
| day53 | 55087 | 54386 | 53044 |
| day54 | 54993 | 54261 | 52856 |
| day55 | 54901 | 54136 | 52668 |
| day56 | 54810 | 54013 | 52480 |
| day57 | 54715 | 53885 | 52285 |
| day58 | 54612 | 53749 | 52083 |
| day59 | 54522 | 53624 | 51886 |
| day60 | 54419 | 53487 | 51683 |
| day61 | 54329 | 53360 | 51481 |

|  | **Government expenditure** | | |
| --- | --- | --- | --- |
|  | Vaccine supplies increasing per day: 0.5% | Vaccine supplies increasing per day: 1% | Vaccine supplies increasing per day: 1.8% |
| day1 | 3370640000000 | 3370640000000 | 3370640000000 |
| day2 | 3370660000000 | 3370660000000 | 3370660000000 |
| day3 | 3370530000000 | 3370530000000 | 3370530000000 |
| day4 | 3370320000000 | 3370320000000 | 3370330000000 |
| day5 | 3370220000000 | 3370220000000 | 3370220000000 |
| day6 | 3370080000000 | 3370090000000 | 3370090000000 |
| day7 | 3369920000000 | 3369930000000 | 3369930000000 |
| day8 | 3369780000000 | 3369780000000 | 3369790000000 |
| day9 | 3369580000000 | 3369590000000 | 3369600000000 |
| day10 | 3369640000000 | 3369650000000 | 3369660000000 |
| day11 | 3369480000000 | 3369500000000 | 3369520000000 |
| day12 | 3369500000000 | 3369520000000 | 3369540000000 |
| day13 | 3369350000000 | 3369370000000 | 3369410000000 |
| day14 | 3369340000000 | 3369370000000 | 3369410000000 |
| day15 | 3369220000000 | 3369250000000 | 3369300000000 |
| day16 | 3369300000000 | 3369330000000 | 3369390000000 |
| day17 | 3369340000000 | 3369380000000 | 3369440000000 |
| day18 | 3369260000000 | 3369300000000 | 3369370000000 |
| day19 | 3369080000000 | 3369130000000 | 3369210000000 |
| day20 | 3368970000000 | 3369020000000 | 3369110000000 |
| day21 | 3368860000000 | 3368920000000 | 3369030000000 |
| day22 | 3368950000000 | 3369020000000 | 3369130000000 |
| day23 | 3368820000000 | 3368890000000 | 3369020000000 |
| day24 | 3368800000000 | 3368880000000 | 3369020000000 |
| day25 | 3368630000000 | 3368720000000 | 3368870000000 |
| day26 | 3368720000000 | 3368810000000 | 3368980000000 |
| day27 | 3368660000000 | 3368760000000 | 3368950000000 |
| day28 | 3368730000000 | 3368840000000 | 3369040000000 |
| day29 | 3368760000000 | 3368880000000 | 3369100000000 |
| day30 | 3368670000000 | 3368800000000 | 3369040000000 |
| day31 | 3368500000000 | 3368640000000 | 3368890000000 |
| day32 | 3368320000000 | 3368470000000 | 3368740000000 |
| day33 | 3368230000000 | 3368390000000 | 3368680000000 |
| day34 | 3368050000000 | 3368220000000 | 3368530000000 |
| day35 | 3368020000000 | 3368200000000 | 3368530000000 |
| day36 | 3368030000000 | 3368220000000 | 3368580000000 |
| day37 | 3368020000000 | 3368230000000 | 3368610000000 |
| day38 | 3367860000000 | 3368070000000 | 3368470000000 |
| day39 | 3367660000000 | 3367890000000 | 3368320000000 |
| day40 | 3367720000000 | 3367960000000 | 3368410000000 |
| day41 | 3367780000000 | 3368030000000 | 3368510000000 |
| day42 | 3367830000000 | 3368090000000 | 3368600000000 |
| day43 | 3367750000000 | 3368030000000 | 3368570000000 |
| day44 | 3367800000000 | 3368090000000 | 3368660000000 |
| day45 | 3367640000000 | 3367940000000 | 3368540000000 |
| day46 | 3367660000000 | 3367980000000 | 3368610000000 |
| day47 | 3367510000000 | 3367850000000 | 3368510000000 |
| day48 | 3367330000000 | 3367680000000 | 3368370000000 |
| day49 | 3367320000000 | 3367690000000 | 3368420000000 |
| day50 | 3367140000000 | 3367530000000 | 3368290000000 |
| day51 | 3367100000000 | 3367500000000 | 3368310000000 |
| day52 | 3367020000000 | 3367440000000 | 3368280000000 |
| day53 | 3367090000000 | 3367530000000 | 3368410000000 |
| day54 | 3367170000000 | 3367620000000 | 3368550000000 |
| day55 | 3366980000000 | 3367450000000 | 3368420000000 |
| day56 | 3366940000000 | 3367430000000 | 3368440000000 |
| day57 | 3366950000000 | 3367460000000 | 3368520000000 |
| day58 | 3366790000000 | 3367310000000 | 3368420000000 |
| day59 | 3366760000000 | 3367310000000 | 3368460000000 |
| day60 | 3366820000000 | 3367380000000 | 3368580000000 |
| day61 | 3366860000000 | 3367440000000 | 3368690000000 |

**SCENARIO C**

|  | **Total infection** | | |  |  |
| --- | --- | --- | --- | --- | --- |
|  | Base(8%,0.5%) | C1(2%,1%) | C2(2%,1.8%) | C3(20%,1%) | C4(20%,1.8%) |
| day1 | 7766520 | 7766520 | 7766520 | 7766520 | 7766520 |
| day2 | 7749020 | 7760060 | 7760060 | 7726940 | 7726940 |
| day3 | 7717530 | 7738470 | 7737980 | 7674790 | 7674300 |
| day4 | 7727340 | 7759670 | 7758120 | 7660070 | 7658530 |
| day5 | 7715320 | 7757830 | 7754690 | 7625020 | 7621890 |
| day6 | 7718610 | 7771140 | 7765740 | 7604480 | 7599120 |
| day7 | 7708570 | 7771990 | 7763660 | 7567880 | 7559650 |
| day8 | 7739130 | 7813950 | 7801980 | 7569720 | 7557920 |
| day9 | 7733590 | 7817690 | 7801410 | 7538600 | 7522600 |
| day10 | 7719480 | 7811630 | 7790220 | 7500000 | 7479000 |
| day11 | 7701500 | 7801900 | 7774620 | 7456010 | 7429320 |
| day12 | 7685540 | 7793080 | 7759100 | 7414900 | 7381730 |
| day13 | 7664420 | 7779850 | 7738270 | 7365880 | 7325410 |
| day14 | 7615640 | 7736450 | 7686670 | 7293210 | 7244870 |
| day15 | 7600750 | 7728460 | 7669160 | 7249450 | 7192040 |
| day16 | 7570440 | 7703550 | 7634130 | 7192380 | 7125350 |
| day17 | 7548240 | 7686690 | 7605970 | 7141770 | 7064050 |
| day18 | 7480850 | 7621370 | 7529130 | 7052010 | 6963320 |
| day19 | 7448170 | 7592180 | 7486620 | 6991600 | 6890370 |
| day20 | 7410550 | 7556700 | 7436820 | 6927360 | 6812630 |
| day21 | 7367900 | 7515010 | 7379820 | 6858920 | 6729770 |
| day22 | 7335050 | 7483910 | 7331840 | 6796670 | 6651800 |
| day23 | 7269230 | 7417290 | 7248370 | 6706440 | 6545790 |
| day24 | 7223880 | 7370890 | 7183320 | 6634530 | 6456470 |
| day25 | 7201520 | 7348080 | 7139310 | 6580850 | 6383180 |
| day26 | 7148670 | 7292560 | 7063010 | 6501650 | 6284660 |
| day27 | 7087940 | 7227680 | 6975820 | 6415260 | 6177480 |
| day28 | 7050780 | 7187220 | 6910470 | 6347290 | 6086650 |
| day29 | 6988140 | 7120060 | 6818000 | 6255830 | 5972040 |
| day30 | 6928320 | 7054980 | 6726180 | 6166690 | 5858510 |
| day31 | 6856870 | 6976740 | 6620620 | 6068150 | 5734940 |
| day32 | 6780890 | 6892830 | 6508200 | 5965690 | 5606330 |
| day33 | 6719070 | 6822710 | 6406900 | 5874400 | 5486610 |
| day34 | 6676200 | 6771150 | 6321240 | 5798700 | 5379950 |
| day35 | 6594050 | 6678780 | 6196450 | 5689490 | 5241320 |
| day36 | 6538310 | 6612770 | 6093440 | 5600400 | 5119090 |
| day37 | 6485940 | 6548780 | 5990810 | 5514950 | 4998770 |
| day38 | 6410770 | 6460290 | 5864390 | 5411210 | 4860430 |
| day39 | 6346040 | 6381690 | 5743980 | 5313710 | 4725330 |
| day40 | 6283410 | 6304430 | 5622800 | 5217010 | 4589370 |
| day41 | 6199190 | 6204420 | 5479770 | 5102560 | 4436390 |
| day42 | 6128470 | 6116880 | 5345560 | 4998550 | 4290600 |
| day43 | 6050950 | 6021830 | 5204140 | 4889700 | 4140190 |
| day44 | 5952600 | 5904880 | 5042090 | 4764310 | 3974070 |
| day45 | 5858100 | 5790780 | 4879710 | 4640400 | 3806980 |
| day46 | 5794920 | 5706610 | 4739690 | 4539620 | 3656610 |
| day47 | 5719520 | 5609670 | 4587480 | 4428720 | 3497100 |
| day48 | 5618750 | 5486520 | 4412040 | 4298860 | 3320460 |
| day49 | 5528480 | 5373450 | 4245110 | 4178120 | 3151620 |
| day50 | 5456710 | 5276680 | 4086210 | 4068270 | 2987050 |
| day51 | 5374330 | 5168800 | 3917220 | 3950280 | 2815460 |
| day52 | 5276670 | 5045250 | 3735010 | 3821960 | 2634560 |
| day53 | 5188270 | 4929590 | 3556430 | 3699180 | 2456160 |
| day54 | 5095500 | 4808500 | 3371080 | 3572190 | 2272540 |
| day55 | 5003790 | 4687730 | 3184950 | 3446180 | 2088730 |
| day56 | 4914450 | 4568340 | 2998030 | 3321940 | 1903960 |
| day57 | 4820810 | 4443500 | 2804200 | 3193220 | 1713920 |
| day58 | 4718930 | 4310210 | 2603260 | 3058890 | 1519020 |
| day59 | 4630390 | 4188070 | 2407000 | 2932510 | 1326510 |
| day60 | 4528970 | 4053950 | 2204410 | 2798440 | 1132210 |
| day61 | 4440130 | 3929370 | 2002550 | 2670470 | 935584 |

|  |  | **Death (per day)** | | |  |
| --- | --- | --- | --- | --- | --- |
|  | Base(8%,0.5%) | C1(2%,1%) | C2(2%,1.8%) | C3(20%,1%) | C4(20%,1.8%) |
| day1 | 1212 | 1212 | 1212 | 1212 | 1212 |
| day2 | 1206 | 1207 | 1207 | 1202 | 1202 |
| day3 | 1251 | 1254 | 1254 | 1244 | 1244 |
| day4 | 1218 | 1223 | 1223 | 1207 | 1207 |
| day5 | 1354 | 1362 | 1361 | 1339 | 1338 |
| day6 | 1343 | 1352 | 1351 | 1323 | 1322 |
| day7 | 1181 | 1191 | 1190 | 1160 | 1159 |
| day8 | 1150 | 1161 | 1159 | 1125 | 1123 |
| day9 | 1271 | 1285 | 1282 | 1239 | 1236 |
| day10 | 1360 | 1376 | 1372 | 1321 | 1317 |
| day11 | 1241 | 1258 | 1253 | 1202 | 1197 |
| day12 | 1285 | 1303 | 1298 | 1240 | 1235 |
| day13 | 1214 | 1232 | 1226 | 1167 | 1160 |
| day14 | 1289 | 1309 | 1301 | 1234 | 1226 |
| day15 | 1295 | 1317 | 1307 | 1235 | 1225 |
| day16 | 1317 | 1340 | 1328 | 1251 | 1240 |
| day17 | 1221 | 1243 | 1230 | 1155 | 1142 |
| day18 | 1321 | 1346 | 1330 | 1246 | 1230 |
| day19 | 1300 | 1325 | 1307 | 1220 | 1203 |
| day20 | 1253 | 1278 | 1258 | 1172 | 1152 |
| day21 | 1228 | 1252 | 1230 | 1143 | 1121 |
| day22 | 1220 | 1244 | 1219 | 1130 | 1106 |
| day23 | 1083 | 1105 | 1080 | 999 | 975 |
| day24 | 1195 | 1219 | 1188 | 1097 | 1068 |
| day25 | 1152 | 1175 | 1142 | 1053 | 1021 |
| day26 | 1223 | 1248 | 1208 | 1112 | 1075 |
| day27 | 1064 | 1085 | 1047 | 963 | 927 |
| day28 | 1100 | 1122 | 1078 | 990 | 950 |
| day29 | 1104 | 1125 | 1077 | 988 | 943 |
| day30 | 1087 | 1107 | 1056 | 968 | 919 |
| day31 | 1177 | 1198 | 1137 | 1042 | 985 |
| day32 | 1190 | 1210 | 1142 | 1047 | 984 |
| day33 | 1116 | 1133 | 1064 | 976 | 911 |
| day34 | 1099 | 1115 | 1041 | 954 | 886 |
| day35 | 998 | 1011 | 938 | 861 | 793 |
| day36 | 1007 | 1018 | 938 | 862 | 788 |
| day37 | 976 | 986 | 902 | 830 | 752 |
| day38 | 1007 | 1014 | 921 | 850 | 763 |
| day39 | 1033 | 1039 | 935 | 865 | 769 |
| day40 | 1071 | 1075 | 959 | 889 | 782 |
| day41 | 1034 | 1034 | 914 | 851 | 739 |
| day42 | 974 | 972 | 849 | 794 | 682 |
| day43 | 920 | 915 | 791 | 743 | 629 |
| day44 | 885 | 878 | 750 | 708 | 591 |
| day45 | 913 | 903 | 761 | 723 | 593 |
| day46 | 1021 | 1005 | 835 | 800 | 644 |
| day47 | 940 | 922 | 754 | 728 | 574 |
| day48 | 996 | 972 | 782 | 762 | 588 |
| day49 | 936 | 909 | 718 | 707 | 533 |
| day50 | 840 | 813 | 629 | 626 | 460 |
| day51 | 878 | 844 | 640 | 645 | 460 |
| day52 | 875 | 837 | 620 | 634 | 437 |
| day53 | 890 | 846 | 610 | 634 | 421 |
| day54 | 826 | 779 | 546 | 579 | 368 |
| day55 | 758 | 710 | 483 | 522 | 316 |
| day56 | 794 | 738 | 484 | 536 | 307 |
| day57 | 786 | 725 | 457 | 521 | 279 |
| day58 | 782 | 715 | 431 | 507 | 252 |
| day59 | 799 | 723 | 415 | 506 | 229 |
| day60 | 694 | 622 | 338 | 429 | 173 |
| day61 | 662 | 586 | 298 | 398 | 139 |

|  | **Total recovered** | | | |  |
| --- | --- | --- | --- | --- | --- |
|  | Base(8%,0.5%) | C1(2%,1%) | C2(2%,1.8%) | C3(20%,1%) | C4(20%,1.8%) |
| day1 | 70796700 | 70796700 | 70796700 | 70796700 | 70796700 |
| day2 | 72750100 | 72750100 | 72750100 | 72750100 | 72750100 |
| day3 | 74714900 | 74724300 | 74738900 | 74723700 | 74738400 |
| day4 | 76670800 | 76698900 | 76743200 | 76697500 | 76741800 |
| day5 | 78644400 | 78700800 | 78790400 | 78697900 | 78787500 |
| day6 | 80610300 | 80704800 | 80855400 | 80700200 | 80850800 |
| day7 | 82617400 | 82759900 | 82987900 | 82752500 | 82980500 |
| day8 | 84600800 | 84801200 | 85123300 | 84791300 | 85113400 |
| day9 | 86605800 | 86874200 | 87307700 | 86860900 | 87294400 |
| day10 | 88615000 | 88961800 | 89524200 | 88944700 | 89507200 |
| day11 | 90647200 | 91082900 | 91792400 | 91061100 | 91770700 |
| day12 | 92675000 | 93209900 | 94085200 | 93183600 | 94058900 |
| day13 | 94736200 | 95381500 | 96441500 | 95349000 | 96409000 |
| day14 | 96807900 | 97574400 | 98838700 | 97535000 | 98799300 |
| day15 | 98878300 | 99776900 | 101266000 | 99730700 | 101219000 |
| day16 | 100961000 | 102003000 | 103736000 | 101949000 | 103683000 |
| day17 | 103049000 | 104246000 | 106245000 | 104184000 | 106184000 |
| day18 | 105159000 | 106522000 | 108809000 | 106452000 | 108739000 |
| day19 | 107266000 | 108807000 | 111404000 | 108728000 | 111325000 |
| day20 | 109376000 | 111105000 | 114036000 | 111018000 | 113948000 |
| day21 | 111491000 | 113421000 | 116708000 | 113325000 | 116612000 |
| day22 | 113626000 | 115770000 | 119436000 | 115664000 | 119331000 |
| day23 | 115780000 | 118150000 | 122220000 | 118033000 | 122104000 |
| day24 | 117923000 | 120530000 | 125031000 | 120403000 | 124904000 |
| day25 | 120067000 | 122925000 | 127881000 | 122789000 | 127746000 |
| day26 | 122232000 | 125353000 | 130791000 | 125206000 | 130645000 |
| day27 | 124404000 | 127801000 | 133748000 | 127643000 | 133592000 |
| day28 | 126583000 | 130269000 | 136753000 | 130101000 | 136586000 |
| day29 | 128797000 | 132786000 | 139834000 | 132603000 | 139653000 |
| day30 | 131016000 | 135321000 | 142962000 | 135124000 | 142768000 |
| day31 | 133243000 | 137878000 | 146143000 | 137667000 | 145934000 |
| day32 | 135478000 | 140457000 | 149375000 | 140232000 | 149153000 |
| day33 | 137714000 | 143050000 | 152653000 | 142811000 | 152418000 |
| day34 | 139944000 | 145651000 | 155973000 | 145401000 | 155726000 |
| day35 | 142216000 | 148308000 | 159379000 | 148042000 | 159117000 |
| day36 | 144487000 | 150979000 | 162833000 | 150698000 | 162556000 |
| day37 | 146750000 | 153657000 | 166330000 | 153363000 | 166040000 |
| day38 | 149029000 | 156366000 | 169892000 | 156058000 | 169589000 |
| day39 | 151321000 | 159102000 | 173517000 | 158780000 | 173200000 |
| day40 | 153625000 | 161866000 | 177206000 | 161528000 | 176875000 |
| day41 | 155953000 | 164670000 | 180971000 | 164314000 | 180623000 |
| day42 | 158276000 | 167484000 | 184787000 | 167113000 | 184425000 |
| day43 | 160611000 | 170326000 | 188670000 | 169939000 | 188293000 |
| day44 | 162967000 | 173205000 | 192629000 | 172800000 | 192234000 |
| day45 | 165336000 | 176113000 | 196657000 | 175688000 | 196245000 |
| day46 | 167690000 | 179023000 | 200734000 | 178584000 | 200308000 |
| day47 | 170072000 | 181977000 | 204894000 | 181520000 | 204452000 |
| day48 | 172471000 | 184965000 | 209132000 | 184489000 | 208671000 |
| day49 | 174870000 | 187970000 | 213434000 | 187476000 | 212957000 |
| day50 | 177271000 | 190996000 | 217804000 | 190486000 | 217312000 |
| day51 | 179694000 | 194059000 | 222256000 | 193531000 | 221748000 |
| day52 | 182125000 | 197149000 | 226784000 | 196602000 | 226259000 |
| day53 | 184565000 | 200266000 | 231389000 | 199701000 | 230848000 |
| day54 | 187020000 | 203416000 | 236076000 | 202832000 | 235518000 |
| day55 | 189482000 | 206592000 | 240841000 | 205989000 | 240266000 |
| day56 | 191943000 | 209785000 | 245681000 | 209167000 | 245092000 |
| day57 | 194423000 | 213016000 | 250610000 | 212380000 | 250005000 |
| day58 | 196916000 | 216280000 | 255626000 | 215625000 | 255005000 |
| day59 | 199408000 | 219563000 | 260722000 | 218893000 | 260087000 |
| day60 | 201931000 | 222894000 | 265916000 | 222203000 | 265264000 |
| day61 | 204447000 | 226240000 | 271192000 | 225533000 | 270526000 |

|  | **Immuned people** | | |  |  |
| --- | --- | --- | --- | --- | --- |
|  | Base(8%,0.5%) | C1(2%,1%) | C2(2%,1.8%) | C3(20%,1%) | C4(20%,1.8%) |
| day1 | 1274610 | 1274610 | 1274610 | 1274610 | 1274610 |
| day2 | 1242230 | 1248410 | 1258300 | 1248410 | 1258300 |
| day3 | 1285190 | 1298000 | 1318650 | 1298000 | 1318650 |
| day4 | 1277410 | 1296570 | 1327630 | 1296570 | 1327630 |
| day5 | 1325910 | 1352490 | 1395860 | 1352490 | 1395860 |
| day6 | 1334630 | 1368160 | 1423210 | 1368160 | 1423210 |
| day7 | 1335170 | 1375520 | 1442200 | 1375520 | 1442200 |
| day8 | 1345980 | 1393560 | 1472680 | 1393560 | 1472680 |
| day9 | 1383970 | 1440030 | 1533850 | 1440030 | 1533850 |
| day10 | 1373300 | 1436030 | 1541700 | 1436030 | 1541700 |
| day11 | 1385730 | 1456240 | 1575780 | 1456240 | 1575780 |
| day12 | 1389140 | 1467080 | 1600090 | 1467080 | 1600090 |
| day13 | 1386930 | 1472030 | 1618210 | 1472030 | 1618210 |
| day14 | 1405840 | 1499530 | 1661490 | 1499530 | 1661490 |
| day15 | 1381370 | 1480760 | 1653690 | 1480760 | 1653690 |
| day16 | 1397650 | 1505660 | 1694820 | 1505660 | 1694820 |
| day17 | 1399330 | 1514980 | 1718810 | 1514980 | 1718810 |
| day18 | 1418940 | 1543850 | 1765440 | 1543850 | 1765440 |
| day19 | 1435180 | 1569280 | 1808740 | 1569280 | 1808740 |
| day20 | 1446320 | 1589330 | 1846360 | 1589330 | 1846360 |
| day21 | 1439610 | 1589830 | 1861560 | 1589830 | 1861560 |
| day22 | 1431940 | 1589230 | 1875600 | 1589230 | 1875600 |
| day23 | 1449840 | 1617100 | 1923620 | 1617100 | 1923620 |
| day24 | 1477740 | 1656420 | 1985990 | 1656420 | 1985990 |
| day25 | 1451870 | 1635520 | 1976470 | 1635520 | 1976470 |
| day26 | 1506190 | 1705150 | 2076930 | 1705150 | 2076930 |
| day27 | 1494130 | 1699920 | 2086960 | 1699920 | 2086960 |
| day28 | 1493660 | 1707830 | 2113290 | 1707830 | 2113290 |
| day29 | 1493480 | 1716120 | 2140360 | 1716120 | 2140360 |
| day30 | 1506040 | 1739160 | 2186280 | 1739160 | 2186280 |
| day31 | 1523050 | 1767550 | 2239570 | 1767550 | 2239570 |
| day32 | 1534500 | 1789710 | 2285600 | 1789710 | 2285600 |
| day33 | 1538370 | 1803150 | 2321010 | 1803150 | 2321010 |
| day34 | 1526490 | 1798120 | 2332860 | 1798120 | 2332860 |
| day35 | 1538280 | 1821030 | 2381310 | 1821030 | 2381310 |
| day36 | 1559820 | 1855710 | 2445870 | 1855710 | 2445870 |
| day37 | 1587330 | 1897830 | 2521200 | 1897830 | 2521200 |
| day38 | 1600550 | 1923160 | 2575090 | 1923160 | 2575090 |
| day39 | 1596090 | 1927340 | 2601130 | 1927340 | 2601130 |
| day40 | 1604340 | 1946940 | 2648400 | 1946940 | 2648400 |
| day41 | 1622790 | 1979130 | 2713500 | 1979130 | 2713500 |
| day42 | 1601380 | 1962740 | 2712350 | 1962740 | 2712350 |
| day43 | 1627030 | 2004090 | 2791430 | 2004090 | 2791430 |
| day44 | 1641020 | 2031380 | 2851860 | 2031380 | 2851860 |
| day45 | 1652120 | 2055300 | 2908280 | 2055300 | 2908280 |
| day46 | 1628120 | 2035530 | 2903120 | 2035530 | 2903120 |
| day47 | 1672780 | 2101760 | 3021330 | 2101760 | 3021330 |
| day48 | 1630680 | 2059050 | 2983380 | 2059050 | 2983380 |
| day49 | 1666890 | 2115250 | 3089080 | 2115250 | 3089080 |
| day50 | 1655490 | 2111240 | 3107640 | 2111240 | 3107640 |
| day51 | 1693330 | 2170240 | 3219790 | 2170240 | 3219790 |
| day52 | 1681210 | 2165430 | 3238100 | 2165430 | 3238100 |
| day53 | 1699850 | 2200320 | 3316340 | 2200320 | 3316340 |
| day54 | 1697260 | 2207900 | 3354130 | 2207900 | 3354130 |
| day55 | 1721940 | 2251150 | 3446920 | 2251150 | 3446920 |
| day56 | 1736920 | 2282030 | 3521870 | 2282030 | 3521870 |
| day57 | 1751830 | 2313080 | 3598060 | 2313080 | 3598060 |
| day58 | 1757500 | 2332100 | 3656390 | 2332100 | 3656390 |
| day59 | 1713150 | 2284560 | 3610230 | 2284560 | 3610230 |
| day60 | 1766680 | 2367660 | 3771190 | 2367660 | 3771190 |
| day61 | 1730400 | 2330590 | 3741540 | 2330590 | 3741540 |

|  | **Unemployed people** | | |  |  |
| --- | --- | --- | --- | --- | --- |
|  | Base(8%,0.5%) | C1(2%,1%) | C2(2%,1.8%) | C3(20%,1%) | C4(20%,1.8%) |
| day1 | 57706 | 51811 | 51811 | 69495 | 69495 |
| day2 | 57688 | 51809 | 51809 | 69395 | 69395 |
| day3 | 57656 | 51803 | 51803 | 69263 | 69261 |
| day4 | 57665 | 51808 | 51808 | 69225 | 69221 |
| day5 | 57653 | 51808 | 51807 | 69136 | 69128 |
| day6 | 57656 | 51811 | 51810 | 69084 | 69071 |
| day7 | 57646 | 51811 | 51809 | 68991 | 68971 |
| day8 | 57676 | 51821 | 51818 | 68996 | 68966 |
| day9 | 57671 | 51822 | 51818 | 68917 | 68876 |
| day10 | 57656 | 51820 | 51815 | 68819 | 68766 |
| day11 | 57638 | 51818 | 51811 | 68708 | 68640 |
| day12 | 57621 | 51815 | 51807 | 68603 | 68520 |
| day13 | 57600 | 51812 | 51801 | 68479 | 68377 |
| day14 | 57550 | 51801 | 51788 | 68295 | 68173 |
| day15 | 57535 | 51798 | 51783 | 68184 | 68039 |
| day16 | 57504 | 51792 | 51774 | 68040 | 67870 |
| day17 | 57482 | 51787 | 51767 | 67911 | 67715 |
| day18 | 57413 | 51771 | 51747 | 67684 | 67460 |
| day19 | 57380 | 51763 | 51736 | 67531 | 67275 |
| day20 | 57342 | 51754 | 51724 | 67368 | 67078 |
| day21 | 57298 | 51743 | 51709 | 67195 | 66868 |
| day22 | 57265 | 51735 | 51697 | 67037 | 66671 |
| day23 | 57198 | 51718 | 51675 | 66809 | 66403 |
| day24 | 57152 | 51706 | 51659 | 66627 | 66176 |
| day25 | 57129 | 51700 | 51647 | 66491 | 65991 |
| day26 | 57076 | 51686 | 51628 | 66290 | 65741 |
| day27 | 57014 | 51669 | 51606 | 66072 | 65470 |
| day28 | 56976 | 51659 | 51589 | 65900 | 65240 |
| day29 | 56913 | 51642 | 51566 | 65668 | 64950 |
| day30 | 56852 | 51625 | 51542 | 65442 | 64663 |
| day31 | 56779 | 51605 | 51515 | 65193 | 64350 |
| day32 | 56702 | 51584 | 51487 | 64934 | 64024 |
| day33 | 56640 | 51566 | 51461 | 64702 | 63721 |
| day34 | 56596 | 51553 | 51439 | 64511 | 63451 |
| day35 | 56513 | 51529 | 51407 | 64234 | 63101 |
| day36 | 56456 | 51512 | 51381 | 64009 | 62791 |
| day37 | 56403 | 51496 | 51355 | 63792 | 62487 |
| day38 | 56327 | 51473 | 51323 | 63530 | 62137 |
| day39 | 56261 | 51453 | 51292 | 63283 | 61795 |
| day40 | 56198 | 51434 | 51261 | 63038 | 61451 |
| day41 | 56112 | 51408 | 51225 | 62749 | 61063 |
| day42 | 56040 | 51386 | 51191 | 62485 | 60694 |
| day43 | 55962 | 51362 | 51155 | 62210 | 60314 |
| day44 | 55862 | 51332 | 51114 | 61892 | 59893 |
| day45 | 55766 | 51303 | 51073 | 61579 | 59471 |
| day46 | 55702 | 51282 | 51037 | 61324 | 59090 |
| day47 | 55626 | 51257 | 50999 | 61043 | 58686 |
| day48 | 55524 | 51226 | 50954 | 60714 | 58239 |
| day49 | 55432 | 51197 | 50912 | 60409 | 57812 |
| day50 | 55360 | 51172 | 50871 | 60131 | 57396 |
| day51 | 55276 | 51145 | 50829 | 59832 | 56962 |
| day52 | 55177 | 51113 | 50782 | 59507 | 56504 |
| day53 | 55087 | 51084 | 50737 | 59197 | 56052 |
| day54 | 54993 | 51053 | 50690 | 58875 | 55588 |
| day55 | 54901 | 51023 | 50643 | 58556 | 55123 |
| day56 | 54810 | 50992 | 50596 | 58242 | 54655 |
| day57 | 54715 | 50961 | 50546 | 57916 | 54174 |
| day58 | 54612 | 50927 | 50496 | 57576 | 53681 |
| day59 | 54522 | 50896 | 50446 | 57257 | 53194 |
| day60 | 54419 | 50862 | 50395 | 56917 | 52702 |
| day61 | 54329 | 50830 | 50343 | 56593 | 52205 |

|  |  | **Government expenditure** | | |  |
| --- | --- | --- | --- | --- | --- |
|  | Base(8%,0.5%) | C1(2%,1%) | C2(2%,1.8%) | C3(20%,1%) | C4(20%,1.8%) |
| day1 | 3370640000000 | 3370640000000 | 3370640000000 | 3370640000000 | 3370640000000 |
| day2 | 3370660000000 | 3370620000000 | 3370620000000 | 3370760000000 | 3370760000000 |
| day3 | 3370530000000 | 3370440000000 | 3370440000000 | 3370720000000 | 3370720000000 |
| day4 | 3370320000000 | 3370180000000 | 3370190000000 | 3370600000000 | 3370600000000 |
| day5 | 3370220000000 | 3370030000000 | 3370040000000 | 3370590000000 | 3370590000000 |
| day6 | 3370080000000 | 3369850000000 | 3369860000000 | 3370550000000 | 3370550000000 |
| day7 | 3369920000000 | 3369650000000 | 3369650000000 | 3370480000000 | 3370490000000 |
| day8 | 3369780000000 | 3369460000000 | 3369470000000 | 3370430000000 | 3370440000000 |
| day9 | 3369580000000 | 3369210000000 | 3369230000000 | 3370320000000 | 3370330000000 |
| day10 | 3369640000000 | 3369230000000 | 3369250000000 | 3370470000000 | 3370490000000 |
| day11 | 3369480000000 | 3369030000000 | 3369060000000 | 3370410000000 | 3370430000000 |
| day12 | 3369500000000 | 3369010000000 | 3369030000000 | 3370520000000 | 3370540000000 |
| day13 | 3369350000000 | 3368810000000 | 3368850000000 | 3370460000000 | 3370490000000 |
| day14 | 3369340000000 | 3368770000000 | 3368810000000 | 3370540000000 | 3370580000000 |
| day15 | 3369220000000 | 3368600000000 | 3368650000000 | 3370510000000 | 3370550000000 |
| day16 | 3369300000000 | 3368640000000 | 3368690000000 | 3370670000000 | 3370730000000 |
| day17 | 3369340000000 | 3368640000000 | 3368700000000 | 3370800000000 | 3370860000000 |
| day18 | 3369260000000 | 3368520000000 | 3368590000000 | 3370810000000 | 3370880000000 |
| day19 | 3369080000000 | 3368300000000 | 3368390000000 | 3370720000000 | 3370800000000 |
| day20 | 3368970000000 | 3368150000000 | 3368250000000 | 3370700000000 | 3370780000000 |
| day21 | 3368860000000 | 3368010000000 | 3368120000000 | 3370680000000 | 3370770000000 |
| day22 | 3368950000000 | 3368060000000 | 3368180000000 | 3370850000000 | 3370950000000 |
| day23 | 3368820000000 | 3367890000000 | 3368020000000 | 3370800000000 | 3370920000000 |
| day24 | 3368800000000 | 3367840000000 | 3367980000000 | 3370870000000 | 3371000000000 |
| day25 | 3368630000000 | 3367630000000 | 3367800000000 | 3370790000000 | 3370920000000 |
| day26 | 3368720000000 | 3367680000000 | 3367860000000 | 3370950000000 | 3371100000000 |
| day27 | 3368660000000 | 3367590000000 | 3367790000000 | 3370980000000 | 3371140000000 |
| day28 | 3368730000000 | 3367630000000 | 3367840000000 | 3371130000000 | 3371300000000 |
| day29 | 3368760000000 | 3367630000000 | 3367860000000 | 3371240000000 | 3371430000000 |
| day30 | 3368670000000 | 3367510000000 | 3367760000000 | 3371230000000 | 3371440000000 |
| day31 | 3368500000000 | 3367310000000 | 3367570000000 | 3371140000000 | 3371360000000 |
| day32 | 3368320000000 | 3367100000000 | 3367390000000 | 3371040000000 | 3371270000000 |
| day33 | 3368230000000 | 3366980000000 | 3367290000000 | 3371020000000 | 3371270000000 |
| day34 | 3368050000000 | 3366760000000 | 3367100000000 | 3370920000000 | 3371180000000 |
| day35 | 3368020000000 | 3366710000000 | 3367070000000 | 3370960000000 | 3371250000000 |
| day36 | 3368030000000 | 3366700000000 | 3367080000000 | 3371050000000 | 3371350000000 |
| day37 | 3368020000000 | 3366660000000 | 3367080000000 | 3371120000000 | 3371430000000 |
| day38 | 3367860000000 | 3366470000000 | 3366910000000 | 3371020000000 | 3371360000000 |
| day39 | 3367660000000 | 3366260000000 | 3366720000000 | 3370900000000 | 3371260000000 |
| day40 | 3367720000000 | 3366280000000 | 3366780000000 | 3371030000000 | 3371400000000 |
| day41 | 3367780000000 | 3366320000000 | 3366850000000 | 3371150000000 | 3371550000000 |
| day42 | 3367830000000 | 3366350000000 | 3366910000000 | 3371280000000 | 3371690000000 |
| day43 | 3367750000000 | 3366260000000 | 3366850000000 | 3371270000000 | 3371700000000 |
| day44 | 3367800000000 | 3366290000000 | 3366910000000 | 3371380000000 | 3371840000000 |
| day45 | 3367640000000 | 3366110000000 | 3366770000000 | 3371290000000 | 3371760000000 |
| day46 | 3367660000000 | 3366110000000 | 3366810000000 | 3371370000000 | 3371870000000 |
| day47 | 3367510000000 | 3365950000000 | 3366690000000 | 3371290000000 | 3371810000000 |
| day48 | 3367330000000 | 3365750000000 | 3366530000000 | 3371160000000 | 3371710000000 |
| day49 | 3367320000000 | 3365730000000 | 3366550000000 | 3371220000000 | 3371790000000 |
| day50 | 3367140000000 | 3365530000000 | 3366400000000 | 3371100000000 | 3371690000000 |
| day51 | 3367100000000 | 3365480000000 | 3366390000000 | 3371120000000 | 3371740000000 |
| day52 | 3367020000000 | 3365390000000 | 3366350000000 | 3371100000000 | 3371740000000 |
| day53 | 3367090000000 | 3365450000000 | 3366460000000 | 3371230000000 | 3371900000000 |
| day54 | 3367170000000 | 3365520000000 | 3366570000000 | 3371360000000 | 3372060000000 |
| day55 | 3366980000000 | 3365330000000 | 3366430000000 | 3371220000000 | 3371950000000 |
| day56 | 3366940000000 | 3365280000000 | 3366430000000 | 3371240000000 | 3371990000000 |
| day57 | 3366950000000 | 3365290000000 | 3366490000000 | 3371300000000 | 3372080000000 |
| day58 | 3366790000000 | 3365120000000 | 3366380000000 | 3371190000000 | 3372000000000 |
| day59 | 3366760000000 | 3365090000000 | 3366410000000 | 3371210000000 | 3372050000000 |
| day60 | 3366820000000 | 3365140000000 | 3366520000000 | 3371320000000 | 3372190000000 |
| day61 | 3366860000000 | 3365180000000 | 3366620000000 | 3371400000000 | 3372300000000 |
